# Long-term follow-up of donor-derived CD7 CAR T-cell therapy in patients with T-cell acute lymphoblastic leukemia

**DOI:** 10.1186/s13045-023-01427-3

**Published:** 2023-04-05

**Authors:** Yue Tan, Lingling Shan, Liping Zhao, Biping Deng, Zhuojun Ling, Yanlei Zhang, Shuixiu Peng, Jinlong Xu, Jiajia Duan, Zelin Wang, Xinjian Yu, Qinlong Zheng, Xiuwen Xu, Zhenglong Tian, Yibing Zhang, Jiecheng Zhang, Alex H. Chang, Xiaoming Feng, Jing Pan

**Affiliations:** 1 State Key Laboratory of Experimental Hematology, Boren Clinical Translational Center, Department of Hematology, Beijing Gobroad Boren Hospital, Beijing, 100070 China; 2 State Key Laboratory of Experimental Hematology, National Clinical Research Center for Blood Diseases, Haihe Laboratory of Cell Ecosystem, Institute of Hematology & Blood Diseases Hospital, Chinese Academy of Medical Sciences & Peking Union Medical College, Tianjin, 300020 China; 3 Tianjin Institutes of Health Science, Tianjin, 301600 China; 4 Cytology Laboratory, Beijing Gobroad Boren Hospital, Beijing, 100070 China; 5 Department of Hematology, Beijing Gobroad Boren Hospital, Beijing, 100070 China; 6 Shanghai YaKe Biotechnology Ltd., Shanghai, 200438 China; 7 Medical Laboratory, Beijing Gobroad Boren Hospital, Beijing, 100070 China; 8 Gobroad Research Center, Gobroad Medical Group, Beijing, 100070 China; 9 Department of Hospital Management, Gobroad Medical Group, Beijing, 100070 China; 10 Clinical Translational Research Center, Shanghai Pulmonary Hospital, Tongji University School of Medicine, Shanghai, 200438 China; 11 Central Laboratory, Fujian Medical University Union Hospital, Fuzhou, 350001 China

**Keywords:** Hematologic malignancy, T-cell acute lymphoblastic leukemia, Chimeric antigen receptor T-cell therapy, Stem cell transplantation

## Abstract

**Background:**

Donor-derived CD7-directed chimeric antigen receptor (CAR) T cells showed feasibility and early efficacy in patients with refractory or relapsed T-cell acute lymphoblastic leukemia (r/r T-ALL), in a previous phase I trial report, at a median follow-up of 6.3 months. Here we report long-term safety and activity of the therapy after a 2-year follow-up.

**Methods:**

Participants received CD7-directed CAR T cells derived from prior stem cell transplantation (SCT) donors or from HLA-matched new donors after lymphodepletion. The target dose was 1 × 10^6^ (± 30%) CAR T cells per kg of patient weight. The primary endpoint was safety with efficacy secondary. This report focuses on the long-term follow-up and discusses them in the context of previously reported early outcomes.

**Results:**

Twenty participants were enrolled and received infusion with CD7 CAR T cells. After a median follow-up time of 27.0 (range, 24.0–29.3) months, the overall response rate and complete response rate were 95% (19/20 patients) and 85% (17/20 patients), respectively, and 35% (7/20) of patients proceeded to SCT. Six patients experienced disease relapse with a median time-to-relapse of 6 (range, 4.0–10.9) months, and 4 of these 6 patients were found to have lost CD7 expression on tumor cells. Progression-free survival (PFS) and overall survival (OS) rates 24 months after treatment were respectively 36.8% (95% CI, 13.8–59.8%) and 42.3% (95% CI, 18.8–65.8%), with median PFS and OS of respectively 11.0 (95% CI, 6.7–12.5) months and 18.3 (95% CI, 12.5–20.8) months. Previously reported short-term adverse events (< 30 days after treatment) included grade 3–4 cytokine release syndrome (CRS; 10%) and grade 1–2 graft-versus-host disease (GVHD; 60%). Serious adverse events reported > 30 days after treatment included five infections and one grade 4 intestinal GVHD. Despite good CD7 CAR T-cell persistence, non-CAR T and natural killer cells were predominantly CD7-negative and eventually returned to normal levels in about half of the participants.

**Conclusions:**

In this 2-year follow-up analysis, donor-derived CD7 CAR T-cell treatment demonstrated durable efficacy in a subset of patients with r/r T-ALL. Disease relapse was the main cause of treatment failure, and severe infection was a noteworthy late-onset adverse event.

***Trial registration*:**

ChiCTR2000034762.

**Supplementary Information:**

The online version contains supplementary material available at 10.1186/s13045-023-01427-3.

## Background

T-lineage acute lymphoblastic leukemia (T-ALL) is an aggressive malignancy derived from early T cell progenitors and constitutes 10% of childhood ALL and 20% of adult ALL cases [1–3]. Despite recent advances in treating the disease, 30% of T-ALL cases are resistant to or relapse after front-line chemotherapy regimens. Allogeneic stem cell transplantation (SCT) has been recommended as a salvage option for these patients, but only those who can be re-induced to remission are eligible [4]. Overall, the prognosis for patients with relapsed or refractory disease is very dismal due to the lack of new treatment options [5–11].

Following the development of a line of chimeric antigen receptor (CAR) T-cell therapies for B cell leukemias, there is growing interest in developing novel cell-based therapies for T-ALL [12]. However, developing therapeutic CAR T-cell treatments for T-ALL is particularly challenging because most validated CAR targets are also expressed on normal T cells. This results in several obstacles to CAR T-cell therapy in T-ALL, including the risk of tumor contamination, fratricide of CAR T cells, and the depletion of healthy T cells, which may make patients more susceptible to opportunistic infection [13]. Despite these hurdles, recent preclinical findings have prompted the initiation of early clinical trials of CAR T-cell therapies for patients with r/r T-ALL [14, 15].

We previously reported early results from a phase I first-in-human study showing that CD7-directed CAR T cells manufactured from T cells collected from allogeneic donors with retention of CD7 molecules in the endoplasmic reticulum could partially overcome these barriers to treat r/r T-ALL [16]. The primary analysis showed that grade 3–4 cytokine release syndrome (CRS) and grade 1 or 2 graft-versus-host disease (GVHD) were short-term adverse events (AEs) that occurred < 30 days after treatment in 10% and 60% of patients, respectively. At 1 month after treatment, 85% of patients had a minimal-residual-disease-negative complete response [16]. Despite these favorable efficacy and safety findings, the short follow-up period of a median of 6.3 months did not allow adequate assessment of remission durability or of long-term AEs, both of which are critical to ascertain the risk–benefit profile.

Here, we report a protocol prespecified long-term analysis of safety and efficacy outcomes in this cohort at a median follow-up time of 27 months. Long-term pharmacokinetics and change of endogenous lymphocyte subpopulations will also be presented.

## Methods

### Patients and study design

Detailed study procedures for this single-center, single-cohort, open-label, phase I study (ChiCTR2000034762) have been reported (Data Supplement) [16]. Briefly, patients between the ages of 0–70 years who had CD7^+^ r/r T-ALL, Eastern Cooperative Oncology Group Performance Status (ECOG-PS) < 3, and no uncontrollable infections or organ failure were considered eligible. The trial followed the principles of the Declaration of Helsinki, and the protocol was approved by the Institutional Review Board at Beijing Gobroad Boren Hospital. All patients supplied written informed consent.

Patients who had received prior-SCT were infused with CD7 CAR T cells obtained from former SCT donors. Patients who had not undergone previous SCT received CD7 CAR T cells obtained from new donors who also provided stem cells for transplantation after CD7 CAR T-cell treatment. On days -5, -4, and -3 prior to infusion, patients who received prior-SCT donor cells underwent lymphodepletion with cyclophosphamide 250 mg/m^2^/day and fludarabine 30 mg/m^2^/day. Patients who received new donor cells underwent enhanced lymphodepletion with cyclophosphamide 30 mg/kg/day and fludarabine 30 mg/m^2^/day on the same days. CD7 CAR T cells were administered as a single intravenous infusion with a target dose of 1 × 10^6^ (± 30%) cells per kg body weight on day 0. Infusion of a lower dose of 5 × 10^5^ (± 30%) cells per kg body weight was allowed if the CAR T-cell product did not meet the target dose, and these data were also included in the safety and efficacy analyses.

### End points

Dose-limiting toxicities (DLTs) within 21 days and incidence of AEs within 30 days, or from day 30 until final visit, were primary endpoints. The ASTCT Consensus was used to grade CRS and neurotoxicity [17]. The EBMT consensus was used to grade GVHD [18]. Other AEs including infections and hematologic toxicities were graded with reference to CTCAE, Version 5.0. Plasma virus activation was routinely monitored by PCR. More details of AE assessment and management are in Additional file 1.

Overall response rate (ORR) (including complete remission [CR] or complete remission with incomplete blood count recovery [CRi]), as assessed by NCCN guidelines, version 1.2020 [4], progression-free survival (PFS), and overall survival (OS) were secondary endpoints. CAR T-cell pharmacokinetics in peripheral blood (PB) and cerebrospinal fluid (CSF) were also secondary endpoints. The survival and disease remission status continued to be followed after receiving SCT or other new anti-leukemia therapies, but documentation of AEs or pharmacokinetics was discontinued. Additional details about endpoints and assessments were as previously described (Data Supplement) [16].

### Statistical analysis

The size of sample was determined based on clinical considerations. The dose was based on previous experience in CAR T-cell therapies in the center. No dose escalation was conducted due to limited capacity for manufacturing, and patient safe run-in still followed a modified 3 + 3 scheme. Time-to-event analysis was based on Kaplan–Meier method. Endpoints were analyzed in subgroups based on prior-SCT donors or new donors, as well as who received the target dose or a low dose. Additional details were in Additional file 1.

## Results

### Patients

**Fig. 1:**
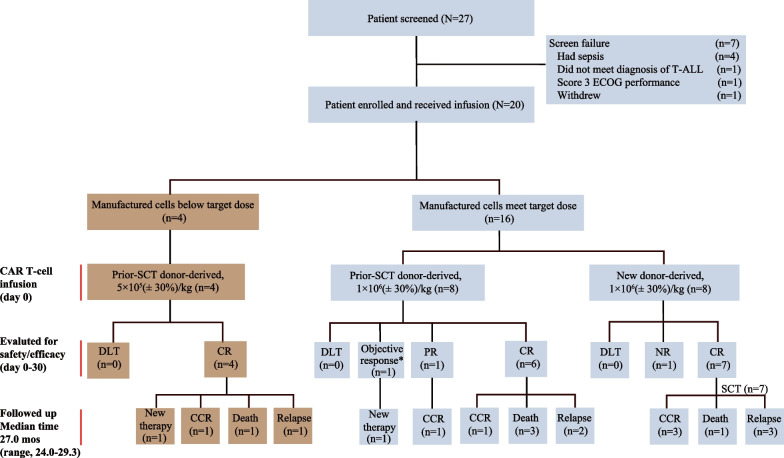
Patient flow diagram. * This patient had an objective response in achieving MRD-negative in BM and CSF and reduction in extramedullary disease. SCT—stem cell transplantation; CR—complete remission; CCR—continuous complete remission; NR—no response; PR—partial remission; DLT—dose limited toxicity; mos—months

As previously reported, 20 participants with r/r T-ALL were enrolled between July 18, 2020 and December 21, 2020, and all patients (100%) received infusion of CD7 CAR T cells (Fig. 1) [16]. At data cutoff on December 20, 2022, the median follow-up time was 27.0 (range, 24.0–29.3) months. Baseline characteristics and early clinical outcomes (median 6.3-month follow-up) for all treated patients, including subgroup analyses of patients based on prior-SCT or on whether the target dose or low dose were administered, have been previously reported (Table 1) [16].

**Table 1:** Baseline characteristics, treatment, and response of individual patients

Pt (no.)	Age/sex (year)	Prior therapies (no.)	BM blasts (%, by morphology)	BM blasts (%, by flow cytometry)	EMDs	CD7^+^ in blasts (%)	CAR T-cell source/Dose (10^5^/kg)	Grade ≥ 3 CRS/onset (mos)	Grade ≥ 3 GVHD/onset (mos)	Grade ≥ 3 Infection/onset (mos)	Response at day 30	Follow-up time (mos)/remission status/live status
***E001***	***12/M***	***3***	**< *5***	***1.35***	***No***	***99.98***	***Prior-SCT/5***	***No***	***No***	***No***	***CR***	***9.0/Loss follow-up***
***E002***	***4/M***	***3***	**< *5***	***1.54***	***No***	***98.80***	***Prior-SCT/5***	***No***	***No***	***Yes/5.5***	***CR***	***5.5/Death of AE***
***E003***	***6/F***	***3***	***9.00***	***18.61***	***No***	***100.00***	***Prior-SCT/5***	***Yes/*< *1***	***No***	***Yes/11–13.3***	***CR***	***28.8/Remission***
*E004*	*6/M*	*4*	< *5*	*0.19*	*No*	*99.95*	*Prior-SCT/10*	*No*	*Yes/8.2*	*Yes/12.3*	*CR*	*12.5/Death of AE*
**E005**	**10/M**	**2**	**18.50**	**11.22**	**Throat/Ileocecal/Inguen**	**99.10**	**New/10**	**No**	**No**	**No**	**CR**	**28.0/Remission**
**E006**	**7/M**	**2**	**80.00**	**41.36**	**No**	**86.42**	**New/10**	**No**	**No**	**No**	**NR**	**0.5/Loss follow-up**
***E007***	***18/M***	***4***	***80.00***	***59.70***	***No***	***100.00***	***Prior-SCT/5***	***No***	***No***	***No***	***CR***	***8.8/Relapse***
*E008*	*23/M*	*4*	< *5*	*0*	*Left neck*	*95.00*^*#*^	*Prior-SCT/10*	*Yes/*< *1*	*No*	*No*	*PR*	*27.1/Remission*
*E009*	*19/M*	*4*	< *5*	*3.86*	*Kidney*	*97.22*	*Prior-SCT/10*	*No*	*No*	*No*	*CR*	*20.8/Relapse*
*E010*	*11/F*	*4*	< *5*	*1.60*	*No*	*81.18*	*Prior-SCT/10*	*No*	*No*	*Yes/5.4–6.8*	*CR*	*6.8/Death of AE*
**E011**	**10/M**	**2**	**< 5**	**0.90**	**No**	**100.00**	**New/10**	**No**	**No**	**No**	**CR**	**26.4//Relapse**
**E012**	**11/M**	**2**	**56.50**	**39.29**	**No**	**100.00**	**New/10**	**No**	**No**	**No**	**CR**	**17.0/Relapse**
*E013*	*17/M*	*4*	*84.00*	*87.99*	*CNS* + *Testicle*	*100.00*	*Prior-SCT/10*	*No*	*No*	*No*	Resp*	*1.8/Loss follow-up*
**E014**	**2/F**	**2**	**< 5**	**6.17**	**No**	**93.80**	**New/10**	**No**	**No**	**No**	**CR**	**14.9/Relapse**
**E015**	**18/M**	**3**	**6.00**	**5.77**	**CNS, Optical nerves**	**90.60**	**New/10**	**No**	**No**	**No**	**CR**	**24.7/Remission**
*E016*	*17/M*	*2*	< *5*	*1.17*	*Mediastinum/Lymphonode*	*100.00*	*Prior-SCT/10*	*No*	*No*	*Yes/8.7*	*CR*	*8.7/Death of AE*
**E017**	**43/F**	**2**	**71.50**	**53.35**	**Breast mass**	**100.00**	**New/10**	**No**	**No**	**No**	**CR**	**1.7/Death of AE**
*E018*	*33/M*	*4*	*89.00*	*16.84*	*No*	*100.00*	*Prior-SCT/10*	*No*	*No*	*No*	*CR*	*24.2/Remission*
**E019**	**5/F**	**2**	**< 5**	**72.40**	**CNS**	**100.00**	**New/10**	**No**	**No**	**No**	**CR**	**24.1/Remission**
*E020*	*10/M*	*3*	< *5*	*0*	*CNS*	*100.00*	*Prior-SCT/10*	*No*	*No*	*No*	*CR*	*18.3/Relapse*

Bold indicates patient who received new donor-derived CAR T cells at 1 × 10^6^ (± 30%)/kg, italics indicates patient who received prior-SCT donor-derived CAR T cells at 1 × 10^6^ (± 30%)/kg, and bolditalics indicates patient who received prior-SCT donor-derived CAR T cells at 5 × 10^5^ (± 30%)/kg

AE—adverse event; Pt—patient; no.—number; F—female; M—male; BM—bone marrow; EMDs—extramedullary diseases; CNS—central nervous system; IHC—immunohistochemistry; CR—complete remission; NR—non-response; CRS—cytokine release syndrome; GVHD—graft-versus-host disease; mos—months; SCT—stem cell transplantation

*This patient achieved objective response (MRD^–^ in BM and CSF, and reduction in EMDs) until out of trial

^#^Determined by immunohistochemistry

### Adverse events

Short-term AEs that had been reported among the 20 treated patients (< 30 days post-infusion), including grade 1–2 CRS in eighteen patients (90%), grade 3–4 CRS in two patients (10%), grade 1–2 neurotoxicity in three patients (15%) and grade 1–2 GVHD in twelve patients (60%). Long-term (> 30 days post-infusion) safety analysis was mainly focused on SAEs of the 12 patients without SCT consolidation (Table 2, Additional file 1: Table S1). The eight patients who received new donor CAR T-cell infusion were either bridged to SCT (*n* = 7) or was off-study (*n* = 1) early after CAR T-cell infusion, and therefore stopped monitoring for AEs. Cytopenias, GVHD and infections were major long-term AEs. A total of six long-term SAEs other than cytopenias were observed, including two that have been previously reported [16].

**Table 2:** Adverse events

	Any time AEs (*n* = 20)	Short-term AEs (*n* = 20)	Long-term AEs (*n* = 12)
CRS			
Any grade	20 (100%)	20 (100%)	0
Grade 3–4	2 (10%)	2 (10%)	0
Grade 5	0	0	0
Neurological events			
Any grade	3 (15%)	3 (15%)	0
Grade 3–4	0	0	0
Grade 5	0	0	0
GVHD			
Any grade	16 (80%)	12 (60%)	7 (58%)
Grade 3–4	1 (5%)	0	1 (8%)
Grade 5	0	0	0
Infection			
Any grade	6 (30%)	3 (15%)	6 (50%)
Grade 3–4	1 (5%)	0	1 (8%)
Grade 5	4 (20%)	0	4 (33%)

Data presented as No, (%) unless otherwise specified

Any time AEs indicate AEs that occurred at any time post-CAR T-cell infusion in all 20 treated patients; short-term AEs indicate AEs that occurred within 30 days post-CAR T-cell infusion in all 20 treated patients; long-term AEs indicated AEs that occurred after 30 days post-CAR T-cell infusion in 12 patients who received prior-SCT donor-derived CAR T cells but did not further undergo a SCT consolidation. Among the 8 patients who received new donor derived CD7 CAR T cells, one was off-study at day 15, and seven proceeded to SCT at about day 30, therefore discontinued monitoring for AEs after 30 days. Cytopenias were described in the text of this report. Other short-term AEs had been previously reported. No other grade 3 or worse AEs occurred in the long-term follow-up

AEs—adverse events; CRS—cytokine release syndrome; GVHD—graft-versus-host disease

Cytopenia, an anticipated side effect of lymphodepletion [19], occurred in all 20 patients within 30 days, of which 100% were grade 3 or higher, and 12 (60%) patients had grade 3 or worse cytopenias before enrollment. Among 12 patients who received prior-SCT donor derived CAR T-cell infusion, all had their cytopenias recovered to grade 2 or lower within 3 months following recombinant human granulocytes/macrophage colony-stimulating factor (rhGM-CSF) administration (*n* = 4) and intravenous infusion of CD34-positive stem cells without preconditioning (*n* = 1) [16]. Three patients had late-onset grade 3 cytopenias at 8, 12.5 and 13 months after CAR infusion, which were suspected to be related to the preceding infection events.

Twelve (60%) of the 20 treated patients developed GVHD within 30 days post-CAR T-cell infusion as previously reported. Late-onset GVHD occurred in 7 (58%) of 12 patients who did not receive SCT consolidation, including six (50%) mild and one (8%) severe cases. Seven patients experienced grade 1 or 2 skin GVHD, with a median onset of 87 days (range, 31–314), manifested as rash maculopapular mostly in the extremities and chest area (< 50% body surface) accompanied by desquamation and pruritus, which were alleviated after treatment with methylprednisolone and ruxolitinib. One of these patients concomitantly developed grade 4 intestinal GVHD at 245–265 days manifested as diarrhea (> 1000 ml/day) and abdominal pain and his symptoms alleviated to grade 1 after giving methylprednisolone, ruxolitinib and mycophenolate mofetil. Another one was a mild lung GVHD at 303–312 days featured by restrictive respiratory dysfunction, which was alleviated after treatment with methylprednisolone, ruxolitinib and cyclosporine. Interestingly, the patient who developed severe GVHD had obviously elevated levels of serum ferritin and lactate dehydrogenase (LDH) compared with those (*n* = 11) without severe GVHD (Additional file 1: Fig. S1). As previously reported, 14 patients received haploidentical donor CAR T cells, and six patients received matched sibling donor (MSD) or matched unrelated donor (MUD) CAR T cells. Patients receiving haploidentical donor cells and patients receiving MSD/MUD cells had a comparable GVHD incidence (9/14 [64%] vs 3/6 [50%] for early GVHD, and 5/8 [63%] vs 2/4 [50%] for late-onset GVHD) and persistence (median 2 days [range, 0–20] vs 1 days [range, 0–12] for early GVHD and median 213 days [range, 0–420] vs 88 days [range, 0–238] for late-onset GVHD) (Additional file 1: Table S2, Fig. S2).

Six (50%) of the 12 patients who did not receive SCT consolidation experienced infections of any grade during the study. Five patients (41.7%) had grade 3 or higher infections with a median onset time of 8.7 (range, 5.4–13.3) months post CAR T-cell infusion. One patient had been previously reported to succumb to pulmonary hemorrhage in the context of fungal pneumonia at 5.5 months. One patient had been previously reported to had mixed CMV and EBV infections at 5.4 months during treatment for his hematochezia in an outer hospital, and in this updated analysis he was recorded to finally die at 6.8 months. The other three severe infections were newly recorded, including a grade 3 CMV encephalitis at 11 months that was resolved after treatment with ganciclovir and anti-CMV immunoglobulin, a grade 5 pulmonary infection at 12.3 months during the immunosuppressant treatment for his intestinal GVHD, and a grade 5 pseudomonas aeruginosa pneumonia at 8.7 months (This patient initially controlled the infection at hospital, but he stopped treatment after discharge leading to disease exacerbation) (Additional file 1:Fig. S3). Of the seven patients with SCT consolidation after CAR T-cell infusion, one (14.3%) was recorded to have a severe infection.

Overall, non-relapse mortality occurred in five (25%) of 20 patients at a median time of 6.8 months (range, 2–12.3) after treatment, including four deaths caused by infections in patients without SCT consolidation and one death caused by engraftment syndrome in a patient after SCT consolidation.

### Efficacy

The ORR in the treated population was previously reported as 95% (95% CI, 76.4–99.1) (*n*=19) at day 30; 85% (95% CI, 64.0–94.8) (*n* = 17) had CR at day 30; 5% (*n* = 1) achieved PR at day 30 and he reached CR at day 45; 5% (*n* = 1) had an objective response at day 30. Of 19 patients who responded, seven patients who received new-donor CAR T cells proceeded to SCT consolidation, two patients withdrew to take alternative therapies at day 55 and 271, and 10 patients did not receive further therapy and were followed for a median time of 27.0 (range, 24.0–29.0) months.

**Fig. 2:**
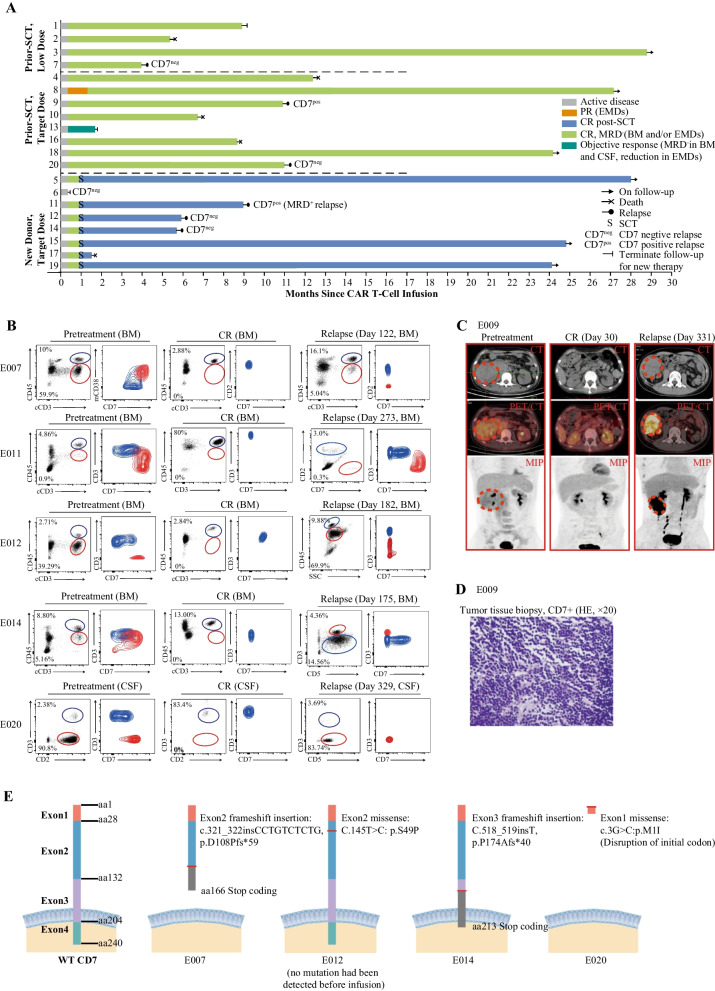
Clinical response and relapse. **A** Swimmer plot shows the clinical response and follow-up of individual patients treated with CD7 CAR T cells, as indicated with different colors in the swimmer lanes. Each bar represents one patient. Patients were listed in different subgroups separated by dotted lines. Patients who were no longer evaluable due to no response (NR) to therapy, loss to follow-up and receiving new therapies other than stem cell transplantation (SCT) are indicated; patients who received SCT still had their survival and relapse information recorded. **B** Flow cytometry plots show CD7 expression on blasts and normal T cells in the BM/CSF of five patients at enrollment, remission, and relapse; contour plots show CD7 expression on blasts (red) and normal T cells (blue) that were merged into the same plot. We had previously reported the flow cytometry plots of pretreatment, CR of all patients and relapse of patient E007. **C** Representative PET/CT of the patient E009 at enrollment, remission, and relapse in extramedullary lesions (enrollment and remission figures had been shown in our previous report). Patient E009 had a kidney lesion, and achieved complete remission at day 30 and relapsed at day 331 post CD7 CAR T cell infusion. **D** The hematoxylin–eosin and other HIC markers (CD1a^−^, CD3^+^, CD4^+^, CD7^+^, CD8^−^, CD33^−^, CD34^−^, CD99^+^) staining of a tissue specimen taken from the tumor tissue in perinephric region shows blasts of patient E009 CD7-positive relapse after CD7 CAR T cell infusion (original magnification × 20). **E** Schematic of the WT and mutated CD7 protein predicted based on next-generation sequencing of DNA extracted from the sample of four CD7-negative relapsed patients; exons and amino acids are numbered. The mutation of patient E007 had been reported previously. CAR—chimeric antigen receptor; CR—complete remission; PR—partial remission; EMDs— extramedullary diseases; MRD—minimal residual disease; BM—bone marrow; CSF—cerebrospinal fluid; CT—computed tomography; PET/CT—position-emission tomography; MIP—maximum intensity projection; HE—hematoxylin–eosin; aa—amino acid; WT—wild-type

By data cutoff date, of 10 patients who did not receive further treatment, three were in remission status, three relapsed (two CD7-negative marrow disease [including a previously reported one], and one CD7-positive extramedullary disease), and four succumbed to infection; Of seven (37%) patients who underwent SCT consolidation, three maintained remission, three relapsed (two CD7-negative marrow disease, and one CD7-positive marrow disease), and one patient died of transplant-related complications (Fig. 2A–D). A total of six patients (33.3% of CR patients) had a relapse, with a median time of 6 (range, 4–11) months post infusion. Next-generation sequencing (NGS) revealed two frameshift and two missense mutations in CD7 gene in specimens from four CD7-negative relapse patients (Fig. 2E). One of them (E012) also performed CD7 sequencing on pretreatment tumor samples, but no mutation was found.

**Fig. 3:**
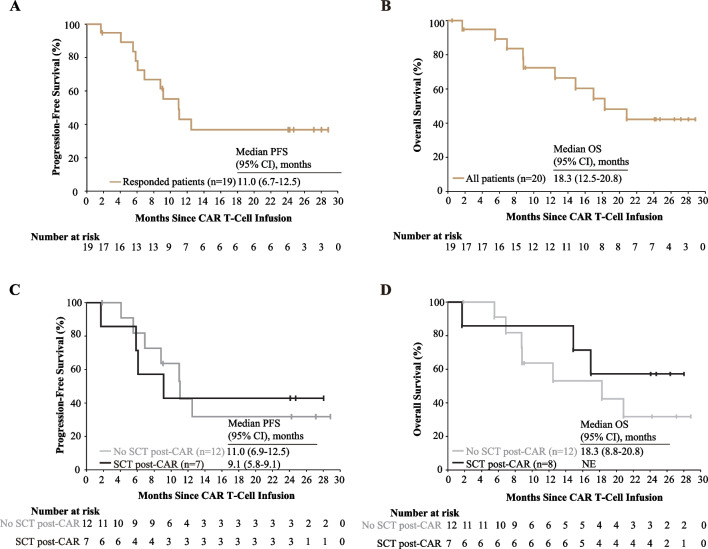
Progression-free survival and overall survival. **A**, **B** Kaplan–Meier estimates of Progression-free survival of 19 responded patients (**A**) and overall survival of all 20 treated patients (**B**). **C**, **D** Kaplan–Meier estimates of progression-free survival of 19 responded patients (**C**), and overall survival of all 20 treated patients (**D**), according to whether or not a subsequent SCT consolidation is conducted. CAR—chimeric antigen receptor; NE—not estimable; SCT—stem cell transplantation; PFS—progression-free survival; OS—overall survival; CI— confidence interval

Median PFS and duration of response (DOR) were respectively 11.0 (range, 6.9–12.5) months and 10.5 (range, 6.4–12.0) months among the 19 responders, and median OS was 18.3 (range, 12.5–20.8) months. The 2-year PFS rate of the 19 responders and OS rate of all 20 treated patients were 36.8% (95% CI, 13.8–59.8%) and 42.3% (95% CI, 18.8–65.8%), respectively (Fig. 3A, B and Additional file 1: Fig. S4A). Post hoc analyses of PFS, DOR, and OS were conducted comparing patients according to whether they received SCT consolidation. For patients without SCT consolidation, 2-year PFS and OS rates were 31.8% and 35%, respectively, and the median PFS and OS were 11.0 (range, 6.9–12.5) months and 18.3 (range, 8.8–20.8) months, respectively. For patients with SCT consolidation, 2-year PFS and OS rates were 42.9% and 58%, respectively, and median PFS was 9.1 (range, 5.8–9.1) months. Median OS was not reached by study endpoint in the subgroup of patients who received SCT consolidation (Fig. 3C, D and Additional file 1: Fig. S4B). Statistical comparison was not conducted owing to the small sample size of the subgroups.

### Pharmocokinetics

**Fig. 4:**
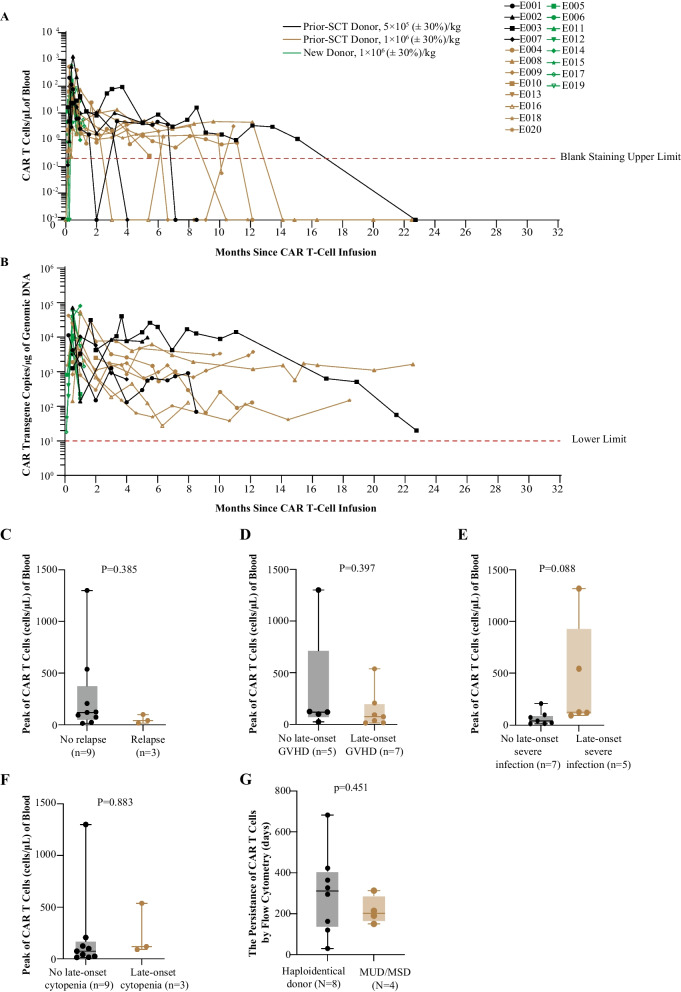
CAR T-cell expansion and persistence. **A** Kinetics of CAR T cells in peripheral blood of individual patients, as measured by flow cytometry; the red dotted line indicates the blank staining upper limit (blank staining with isotype control antibody were included in each sample analysis, and ranged from 0 to 0.02%); data were recorded until the cut-off date or the time point that discontinued follow-up; different types of symbols indicate different patients, and lines of different colors indicate different subgroups. **B** Kinetics of CAR vector transgene copies per microgram of genomic DNA of peripheral blood mononuclear cells in individual patients, as measured by quantitative PCR; the dotted line indicates the lower limit of quantitation for this assay; data were recorded until the cut-off date or the time point that discontinued follow-up; different types of symbols indicate different patients, and lines of different colors indicate different groups. **C**–**F** The peak numbers of CAR T cells in the peripheral blood of the 12 patients without SCT consolidation according to long-term response (**C**) or adverse events (**D**–**F**). **G** CAR T-cell persistence determined by flow cytometry according receiving haploidentical or MSD/MUD cells. The longest horizontal bars indicate the median values, the gray and light orange boxes indicate values that range from 25 to 75th percentile, different color of symbols indicate patients from different subgroups, and the whiskers extend from the minimum to the maximum values. The *P* value is based on unpaired student’s *t*-tests. CAR—chimeric antigen receptor; PCR—polymerase chain reaction; SCT—stem cell transplantation; MSD—matched sibling donor; MUD—matched unrelated donor; GVHD—graft-versus-host disease

The short-term kinetics of CAR T-cell infusion was reported previously. CD7 CAR T cells reached peak levels in blood between days 7–14 after treatment, with a median concentration of 83.70 (range, 5.45–1,300.00) cells/μl. One patient who was discharged from the study early and seven patients who received SCT consolidation did not have long-term monitoring with CAR T cells. Among 12 patients without SCT consolidation, CAR T cells could be detectable by flow cytometry in 100% (seven of seven evaluable patients) and 50% (two of four evaluable patients) at 6 and 12 months post-CAR T-cell infusion (Fig. 4A). CD7 CAR T cells were still detectable by flow cytometry in two patients before their CD7^−^ relapses and undetectable by flow cytometry in a patient before his CD7^+^ relapse. All evaluable patients had CAR transgenes detectable by quantitative polymerase chain reaction (PCR) at the time of the last assessments, and the median duration of CAR T-cell persistence at flow cytometric level in the 12 patients without SCT consolidation was 255 (range, 30–682) days (Fig. 4B). Of note, peak CAR T-cell counts did not significantly differ between patient subgroups according to long-term remission or relapse. The incidence of late-onset severe infection, GVHD or cytopenias was not significantly associated with a higher peak CAR T-cell counts (Fig. 4C–F). There was no difference in the persistence of CAR T cells between patients receiving haploidentical and MSD/MUD cells (Fig. 4G).

### T-cell aplasia

**Fig. 5:**
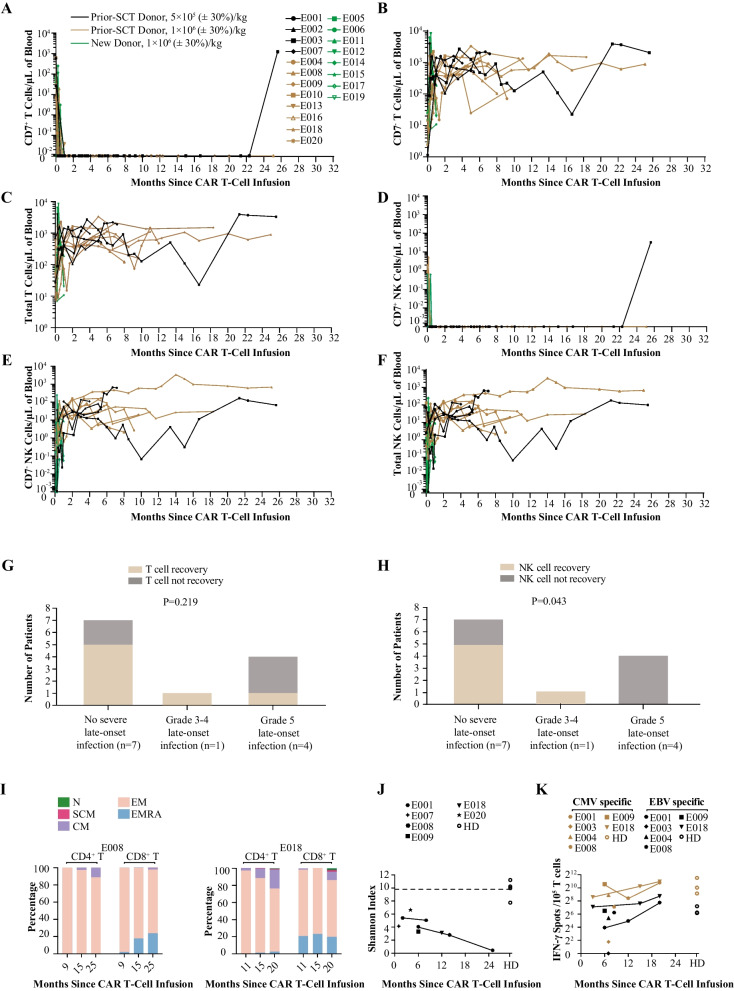
T-cell aplasia. **A**–**F** Kinetics of CD7^+^CD3^+^ T cells (**A**), CD7^−^CD3^+^ T cells (**B**), total CD3^+^ T cells (**C**), CD7^+^CD16^+^CD56^+^ NK cells (**D**), CD7^−^CD16^+^CD56^+^ NK cells (**E**), and total CD16^+^CD56^+^ NK cells (**F**) in peripheral blood of all treated patients, as measured by flow cytometry; CAR T and leukemia cells were excluded in this analysis; data were recorded until the cut-off date or the time point that discontinued follow-up; different types of symbols indicate different patients, and lines of different colors indicate different subgroups. **G**, **H** Association of T-cell recovery (**G**) or NK-cell recovery (**H**) with late-onset severe infection. The *P* value is based on Chi-square tests. **I** Percentage stacked bar charts show kinetics of subset compositions among CD3^+^CD4^+^ and CD3^+^CD8^+^ T cells in peripheral blood of two patients, as measured by flow cytometry. **J** T cell receptor sequencing data, presented as Shannon indexes from post-CD7 CAR T-cell therapy patients and healthy donors (some short-term results had been shown in our previous report, including E001 at month 2, E007 at month 1, E008 at month 6 and E009 at month 6); dashed line indicates median of normal values. **K** IFN-γ expressing T cells in PBMCs from post-CD7 CAR T-cell therapy patients and healthy donors after stimulation with CMV or EBV peptides (some short-term results had been shown in our previous reports, including E001 at month 8, E003 at month 7, E004 at month 7, E008 at month 6, E009 at month 6 and E018 at month 3), as determined by ELISPOT assay. CAR—chimeric antigen receptor; CMV—cytomegalovirus; EBV—Epstein-Barr virus; IFN—interferon; N—CD45RA^+^CD62L^+^CD95^−^ naïve T cells; SCM—CD45RA^+^CD62L^+^CD95^+^ stem cell-like memory T cells; CM —CD45RA^−^CD62L^+^ central memory T cells; EM—CD45RA^−^CD62L^−^ effector memory; EMRA—CD45RA^+^CD62L^−^ effector memory cell re-expressing CD45RA; PBMC—peripheral blood mononuclear cells; HD—healthy donor

In all treated patients, non-CAR CD7^+^ T and NK cells were rapidly depleted within 15 days of CD7 CAR T-cell infusion, which was accompanied by an elevated count of CD7^−^ T and NK cells (Fig. 5A–F and Additional file 1: Fig. S5). Patients who received prior-SCT donor CAR T cells had complete chimerism status in peripheral blood T cells after infusion, whereas patients with new donor CAR T cells had complete or mixed chimerism after infusion (Additional file 1: Table S3). T and NK cells were monitored with long-term in the 12 patients who did not receive SCT consolidation. CD7^+^ T and NK cells remained undetectable in all patients until the last visit, excepting one patient who had recovery of CD7^+^ T and NK cells 25.6 months post-infusion following loss of flow-cytometry-detectable CD7 CAR T cells at 22.7 months. The number of CD7^−^ T,  total T and NK cells progressively increased, and by the last visit, total T-cell counts recovered to normal levels in 7 (58%) of 12 patients at a median time of 1.9 (range, 0.4–4.3) months (Fig. 5C). Total NK-cell counts recovered to normal levels in 6 (50%) of 12 patients at a median time of 5.1 (range, 1.9–21.4) months (Fig. 5F). Of the five patients with severe infections, two had T-cell recovery and one had NK-cell recovery, whereas of the 7 patients without severe infection, five had T-cell recovery and five had NK-cell recovery (Fig. 5G, H). All patients (100%) exhibited a low CD4^+^/CD8^+^ T-cell ratio within 1 month, and seven patients (55%) recovered to normal CD4^+^/CD8^+^ T-cell ratio before last visit (Additional file 1: Fig. S6). B-cell recovered in seven patients (> 5% of lymphocytes) between days 25 and 100 as previously reported [16]. In this follow-up study, one additional patient had B-cell recovery at 17 months, and the other four patients remained < 5% B cells among lymphocytes until the last visit, which was suspected to be related to the presence of GVHD.

Our previous short-term analysis showed a lack of naïve subpopulations of non-CAR T cells early after CD7 CAR T-cell infusion, but patients had a partially preserved response to viral and fungal antigen stimulation [16]. Long-term monitoring of T-cell phenotype and function in 2 patients showed that the central memory T-cell subpopulation gradually increased, and low levels of naïve and stem-cell-memory T-cell subpopulations were detectable in 1 of these patients after 15 months (Fig. 5I, Additional file 1: Fig. S7A). TCR diversity in patients after CD7 CAR T-cell infusion remained lower compared to healthy donors (Fig. 5J, Additional file 1: Fig. S7B). However, the response of T cells post-infusion to CMV and EBV antigen stimulation was in a trend of elevation, suggesting certain degree of protective function against these viruses. (Fig. 5K, Additional file 1: Fig. S7C).

## Discussion

This study provides a 2-year follow-up in 20 participants with r/r T-ALL after therapy with CD7-directed CAR T cells originated from donors. We showed durable remissions lasted for more than 24 months in a proportion of treated patients, whereas relapse emerges as a main cause of treatment failure. This research also raised infection and GVHD as major long-term adverse events in patients without SCT consolidation.

The safety analysis in this follow-up study provides further insights into long-term risks of the therapy. Our previous report demonstrated manageable short-term adverse events, including cytopenias, CRS, neurotoxicity and acute GVHD. Here we showed that late-onset GVHD was the most common long-term AEs with 58% incidence among the 12 patients without SCT consolidation. The allogenic origin of T cells was suspected to be a contributing factor in late-onset GVHD, however these complications were mostly mild and all manageable. Interestingly, our results did not show a significant association between human leukocyte antigen (HLA) matching degree with incidence or persistence of GVHD, but due to the small sample this issue needs to be further investigated in future studies. Only one case of severe GVHD was reported, and no severe GVHD occurred beyond 12 months post infusion, suggesting that this AE was generally manageable.

Grade three or worse infections occurred in 42% (5/12) of participants who did not receive SCT consolidation. These severe infections mostly occurred around 6 months to 1 year post infusion, and were suspected to be caused by mixed effects of normal T-cell depletion, cytopenias, and immunosuppressive agents that were used to control GVHD. During the follow-up period, 58% (7/12) of patients who did not underwent SCT consolidation had their non-CAR T cells restored to normal level. The incidence of severe infections seemed to be negatively correlated with T-cell recovery, although not statistically significant. Long-term monitoring of T cells in some patients showed increased central memory subpopulation and good responsiveness to viral antigen stimulation, suggesting that they had some protective effects. Notably, a lower incidence of severe infections (1/7, 14.3%) were observed in patients with SCT consolidation after CAR T-cell infusion, suggesting that early bridging to SCT may reduce the risk of life-threatening infection. For patients who were planning to receive new donor-derived CD7 CAR T cells, we would recommend them to discard this CAR T-cell therapy if subsequent SCT was not feasible. Patients treated with prior-SCT donor-derived CD7 CAR T cells mostly had available donors and were also strongly recommended for SCT consolidation, but most of them declined it for personal reasons. The future effort will be made to arrange most patients to accept SCT after CAR T-cell infusion. For patients who are not eligible for SCT, using a molecular switch to terminate CAR T cells, or early infusion with purified CD34^+^ stem cells to promote T-cell recovery, may be the alternative strategies to reduce the risk of severe infections [20]. Nonetheless, the relationship between T-cell recovery and risk of severe infection, as well as the ways to overcome this challenge, remains to be further investigated.

The treatment produced a median PFS of 11.0 months and a median OS of 18.3 months, and 31.5% (*n* = 6) of the responders achieved responses lasting more than 2 years, including three who did not receive further consolidation. A previous study reported a median OS of 8 months among patients with r/r T-ALL treated with salvage nelarabine treatment [21]. Of the six (33.3%) patients who relapsed, four were CD7-negative relapse and two were CD7-positive relapse. CD19-negative relapse frequently occurred during CD19 CAR T-cell therapy for B-cell malignancies, with various mechanisms including mutation of CD19 gene and evolution from pre-existing CD19-negative subclones [22, 23]. Frameshift or missense mutations were detected in the four CD7-negative relapse patients in our study, suggesting that mutation may be a main cause of CD7 loss in tumor cells. However, due to the lack of sufficient samples, it remains to be determined in future research whether CD7-negative relapse was derived from CD7-negative leukemic (or preleukemic) clones that exist before therapy, or caused by a new mutation during CAR T-cell treatment. We also observed CD7-positive relapse following the loss of CAR T cells (at flow cytometric level) in a patient without SCT consolidation, suggesting that insufficient persistence may also be a cause of relapse. However, the incidence of this antigen-positive relapse is relatively low, consistent with the overall good CAR T-cell persistence that are possibly related to the fine CAR vector design and donor complete chimerism status.

The CD7-negative T cells after CAR T-cell therapy could be derived from endogenous CD7-negative T cells (naturally developed) preexisted in patients, or from the infused T-cell product (naturally developed or caused by endoplasmic reticulum retention of CD7 protein). The eight patients who received new donor CAR T cells obtained complete or mixed chimerism status at 1 month after CAR T-cell infusion, suggesting that their CD7-negative T cells were mostly or partially derived from the T-cell product. The 12 patients who received prior-SCT donor-derived CAR T cells had already achieved complete chimerism after SCT and contain donor stem-cell differentiated CD7-negative T cells, therefore it could not be determined whether their CD7-negative T cells were derived from endogenous T cells or infused T-cell product. The clear dissection of the origin and function of these CD7-negative T cells warrants further analysis.

Differences in study designs and patient characteristics make it difficult to directly compare the results of donor CD7 CAR T-cell therapy with the recent reports of other ongoing CD7-targeted cellular therapies. Genome-edited universal CD7 CAR T cells, autologous nanobody-based CD7 CAR T cells and naturally selected CD7 CAR T cells also showed early efficacy in patients with T-ALL, while CD7-negative relapses were also observed in these studies [16, 24–26]. Fewer severe infection cases were reported in these studies. However, these therapies had a less than 1-year median follow-up or were mostly bridged to SCT early after CAR T-cell infusion, making it difficult to evaluate the risk of infection with the long-term presence of CAR T cells. In contrast, our patients were followed up for more than 2 years, and more than half of them did not receive SCT post CAR T-cell infusion, allowing us to assess the long-term risk of infection. Donor-derived CD7 CAR T cells may be particularly useful, when autologous CD7 CAR T-cell therapy is unavailable due to low quantity or quality of patient’s T cells, or risk of tumor contamination. Indeed, the preparation of autologous T cell for patients with T-ALL in some patients may be very challenging, since the chemotherapy regimen were usually very intense and designed against T-lineage cells [1]. A parallel phase I trial is undertaken in our center to test feasibility of autologous CD7 CAR T cells against T-ALL with the same lentiviral vector, and this will provide further information to compare the application between donor and autologous CD7 CAR T cells [27].

This study has several limitations. Firstly, it was a phase I trial designed to initially explore feasibility and safety with a small sample size. Therefore, a phase II study with a larger sample size is needed to confirm safety and efficacy and to define prognostic factors. In addition, it is the first experience with donor CD7 CAR T cells for T-ALL, with no prior clinical experience, and regimens for managing adverse events need to be further optimized. Also, long-term functionality of non-CAR T cells and the mechanisms of CD7 antigen loss that leads to relapse warrants further investigation.

The main strength of this study is that it presents a first long-term follow-up in patients with T-ALL after CAR T-cell treatment. The durability of responses and new signals of long-term adverse events showed here, as well as previous report of a high early response rate, support that donor-derived CD7 CAR T cells may be a feasible salvage treatment for children or adults with r/r T-ALL. Severe infection appears to the notable side effect associated with this therapy. Early bridging to SCT consolidation has the potential to reduce this risk of infection, and monitoring of immune function and careful prevention and treatment of infection is critical for those patients for whom a subsequent SCT is not feasible. To know more about the benefit and risk profile, a multicenter phase II clinical trial is ongoing, which may provide additional clues for further optimization of this therapy.

## Conclusions

In conclusion, this long-term report initially provides assessment of remission durability and long-term adverse events, both of which are critical to understanding the activity, beneficial effects, and risks of CD7 CAR T-cell therapy, and provides valuable information for future design of CD7-targeted cellular therapy. In our long-term analysis, donor-derived CD7 CAR T-cell treatment demonstrated durable efficacy in a subset of patients with r/r T-ALL. The failure of donor-derived CD7 CAR T-cell treatment in r/r T-ALL may be due to relapse. Additionally, severe infection was a noteworthy late-onset adverse event.

## Supplementary Information

**Additional file 1.** Materials and Methods. **Fig. S1.** Severe GVHD associated with higher LDH and ferritin in blood. **Fig. S2.** The incidence and persistance of GVHD according to the degree of HLA matching. **A** Early GVHD in patients receiving haploidentical donor cells and matched unrelated donor or matched sibling donor cells (MUD/MSD). **B** Late-onset GVHD in patients receiving haploidentical donor cells and matched unrelated donor or matched sibling donor cells (MUD/MSD). **C** Persistence of early GVHD between patients receiving haploidentical donor cells and MUD/MSD cells. **D** Persistence of late-onset GVHD between patients receiving haploidentical donor cells and MUD/MSD cells. **Fig. S3.** EBV and CMV viral load in the plasma. **A** Copy numbers of CMV DNA in plasma of individual patients. **B** Copy numbers of EBV DNA in plasma of individual patients. **Fig. S4.** Kaplan-Meier curves of duration of response after CD7 CAR T-cell therapy. **A** Kaplan-Meier curves of duration of response in all responded patients after CD7 CAR T-cell therapy. **B** Kaplan-Meier curves of duration of response in subgroups according to SCT after CD7 CAR T-cell therapy. **Fig. S5.** Representative staining of T, NK and NKT cells in the peripheral blood of a patient. **Fig. S6.** CD4^+^ and CD8^+^ T cell ratio in the peripheral blood of all patients. **Fig. S7.** Characterization of CD7^-^ T cells from patients post CD7 CAR T-cell therapy. **A** CD45RA, CD62L and CD95 expression on CD4^+^ and CD8^+^ T cells from E018 at month 20 post CD7 CAR T-cell therapy and a healthy donor, as determined by flow cytometry. **B** T cell receptor (TCR) sequencing data, including the sum of the sequencing reads, TCR clones reconstructing from the sequencing reads and shannon entropy as an index of TCR Vβ usage diversity from indicated patients and healthy donors. **C** The number of T cells secreting IFN-γ in PBMCs from indicated patients, and healthy donors, after stimulation with phytohemagglutinin (PHA, as positive control), cytomegalovirus (CMV) or Epstein-Barr virus (EBV) peptide, as determined by IFN-γ ELISPOT assay. **Table S1.** Adverse events of special interest in all 20 treated patients and by subgroups. **Table S2.** The degree of HLA matching and GVHD. **Table S3.** Chimerism status after CAR T-cell infusion in all 20 treated patients.

## Data Availability

The datasets generated and analyzed during the current study are available in the published article and its additional files.

## Abbreviations

AEs: Adverse events
CAR: Chimeric antigen receptor
CRi: Complete remission with incomplete blood count recovery
CR: Complete remission
CRS: Cytokine release syndrome
CSF: Cerebrospinal fluid
DLTs: Dose-limiting toxicities
DOR: Duration of response
ECOG-PS: Eastern Cooperative Oncology Group Performance Status
GVHD: Graft-versus-host disease
LDH: Lactate dehydrogenase
HLA: Human leukocyte antigen
MSD: Matched sibling donor
MUD: Matched unrelated donor
NGS: Next-generation sequencing
NK: Natural killer
ORR: Overall response rate
OS: Overall survival
PB: Peripheral blood
PCR: Polymerase chain reaction
PFS: Progression-free survival
r/r T-ALL: Refractory or relapsed T-cell acute lymphoblastic leukemia
SAEs: Severe adverse events
SCT: Stem cell transplantation

## References

[1] Teachey DT, Pui CH (2019). Comparative features and outcomes between paediatric T-cell and B-cell acute lymphoblastic leukaemia. Lancet Oncol.

[2] Marks DI, Paietta EM, Moorman AV, Richards SM, Buck G, DeWald G (2009). T-cell acute lymphoblastic leukemia in adults: clinical features, immunophenotype, cytogenetics, and outcome from the large randomized prospective trial (UKALL XII/ECOG 2993). Blood.

[3] Jain P, Kantarjian H, Ravandi F, Thomas D, O'Brien S, Kadia T (2014). The combination of hyper-CVAD plus nelarabine as frontline therapy in adult T-cell acute lymphoblastic leukemia and T-lymphoblastic lymphoma: MD Anderson Cancer Center experience. Leukemia.

[4] Brown P, Inaba H, Annesley C, Beck J, Colace S, Dallas M (2020). Pediatric acute lymphoblastic leukemia, version 2.2020, NCCN clinical practice guidelines in oncology. J Natl Compr Cancer Netw.

[5] Thomas DA, Kantarjian H, Smith TL, Koller C, Cortes J, O'Brien S (1999). Primary refractory and relapsed adult acute lymphoblastic leukemia: characteristics, treatment results, and prognosis with salvage therapy. Cancer.

[6] Tavernier E, Boiron JM, Huguet F, Bradstock K, Vey N, Kovacsovics T (2007). Outcome of treatment after first relapse in adults with acute lymphoblastic leukemia initially treated by the LALA-94 trial. Leukemia.

[7] Fielding AK, Richards SM, Chopra R, Lazarus HM, Litzow MR, Buck G (2007). Outcome of 609 adults after relapse of acute lymphoblastic leukemia (ALL); an MRC UKALL12/ECOG 2993 study. Blood.

[8] Oriol A, Vives S, Hernández-Rivas JM, Tormo M, Heras I, Rivas C (2010). Outcome after relapse of acute lymphoblastic leukemia in adult patients included in four consecutive risk-adapted trials by the PETHEMA Study Group. Haematologica.

[9] Gökbuget N, Stanze D, Beck J, Diedrich H, Horst HA, Hüttmann A (2012). Outcome of relapsed adult lymphoblastic leukemia depends on response to salvage chemotherapy, prognostic factors, and performance of stem cell transplantation. Blood.

[10] Spyridonidis A, Labopin M, Schmid C, Volin L, Yakoub-Agha I, Stadler M (2012). Outcomes and prognostic factors of adults with acute lymphoblastic leukemia who relapse after allogeneic hematopoietic cell transplantation. An analysis on behalf of the Acute Leukemia Working Party of EBMT. Leukemia.

[11] Poon LM, Hamdi A, Saliba R, Rondon G, Ledesma C, Kendrick M (2013). Outcomes of adults with acute lymphoblastic leukemia relapsing after allogeneic hematopoietic stem cell transplantation. Biol Blood Marrow Transplant.

[12] Bayón-Calderón F, Toribio ML, González-García S (2020). Facts and Challenges in Immunotherapy for T-cell acute lymphoblastic leukemia. Int J Mol Sci.

[13] Gomes-Silva D, Srinivasan M, Sharma S, Lee CM, Wagner DL, Davis TH (2017). CD7-edited T cells expressing a CD7-specific CAR for the therapy of T-cell malignancies. Blood.

[14] Freiwan A, Zoine JT, Crawford JC, Vaidya A, Schattgen SA, Myers JA (2022). Engineering naturally occurring CD7-T cells for the immunotherapy of hematological malignancies. Blood.

[15] Diorio C, Murray R, Naniong M, Barrera L, Camblin A, Chukinas J (2022). Cytosine base editing enables quadruple-edited allogeneic CART cells for T-ALL. Blood.

[16] Pan J, Tan Y, Wang G, Deng B, Ling Z, Song W (2021). Donor-derived CD7 chimeric antigen receptor T cells for T-cell acute lymphoblastic leukemia: first-in-human, phase I trial. J Clin Oncol.

[17] Lee DW, Santomasso BD, Locke FL, Ghobadi A, Turtle CJ, Brudno JN (2019). ASTCT consensus grading for cytokine release syndrome and neurologic toxicity associated with immune effector cells. Biol Blood Marrow Transplant.

[18] Przepiorka D, Weisdorf D, Martin P, Klingemann HG, Beatty P, Hows J (1995). Consensus conference on acute GVHD grading. Bone Marrow Transplant.

[19] Fried S, Avigdor A, Bielorai B, Meir A, Besser MJ, Schachter J (2019). Early and late hematologic toxicity following CD19 CAR-T cells. Bone Marrow Transplant.

[20] Mullanfiroze K, Lazareva A, Chu J, Williams L, Burridge S, Silva J (2022). CD34+-selected stem cell boost can safely improve cytopenias following CAR T-cell therapy. Blood Adv.

[21] Candoni A, Lazzarotto D, Ferrara F, Curti A, Lussana F, Papayannidis C (2020). Nelarabine as salvage therapy and bridge to allogeneic stem cell transplant in 118 adult patients with relapsed/refractory T-cell acute lymphoblastic leukemia/lymphoma. A CAMPUS ALL study. Am J Hematol.

[22] Orlando EJ, Han X, Tribouley C, Wood PA, Leary RJ, Riester M (2018). Genetic mechanisms of target antigen loss in CAR19 therapy of acute lymphoblastic leukemia. Nat Med.

[23] Rabilloud T, Potier D, Pankaew S, Nozais M, Loosveld M, Payet-Bornet D (2021). Single-cell profiling identifies pre-existing CD19-negative subclones in a B-ALL patient with CD19-negative relapse after CAR-T therapy. Nat Commun.

[24] Zhang M, Chen D, Fu X, Meng H, Nan F, Sun Z (2022). Autologous nanobody-derived fratricide-resistant CD7-CAR T-cell therapy for patients with relapsed and refractory T-cell acute lymphoblastic leukemia/lymphoma. Clin Cancer Res.

[25] Hu Y, Zhou Y, Zhang M, Zhao H, Wei G, Ge W (2022). Genetically modified CD7-targeting allogeneic CAR-T cell therapy with enhanced efficacy for relapsed/refractory CD7-positive hematological malignancies: a phase I clinical study. Cell Res.

[26] Lu P, Liu Y, Yang J, Zhang X, Yang X, Wang H (2022). Naturally selected CD7 CAR-T therapy without genetic manipulations for T-ALL/LBL: first-in-human phase 1 clinical trial. Blood.

[27] Zhao L, Pan J, Tang K, Tan Y, Deng B, Ling Z (2022). (2022) Autologous CD7-targeted CAR T-cell therapy for refractory or relapsed T-cell acute lymphoblastic leukemia/lymphoma. J Clin Oncol.

