# Supplementary material for: Long-term follow-up of donor-derived CD7 CAR T-cell therapy in patients with T-cell acute lymphoblastic leukemia
Source: J Hematol Oncol. 2023 Apr 5;16:34. doi: 10.1186/s13045-023-01427-3 (PMC10074659; doi:10.1186/s13045-023-01427-3)
Supplement: Supplementary file 1 — Additional file 1. Materials and Methods. Fig. S1. Severe GVHD associated with higher LDH and ferritin in blood. Fig. S2. The incidence and persistance of GVHD according to the degree of HLA matching. A Early GVHD in patients receiving haploidentical donor cells and matched unrelated donor or matched sibling donor cells (MUD/MSD). B Late-onset GVHD in patients receiving haploidentical donor cells and matched unrelated donor or matched sibling donor cells (MUD/MSD). C Persistence of early GVHD between patients receiving haploidentical donor cells and MUD/MSD cells. D Persistence of late-onset GVHD between patients receiving haploidentical donor cells and MUD/MSD cells. Fig. S3. EBV and CMV viral load in the plasma. A Copy numbers of CMV DNA in plasma of individual patients. B Copy numbers of EBV DNA in plasma of individual patients. Fig. S4. Kaplan-Meier curves of duration of response after CD7 CAR T-cell therapy. A Kaplan-Meier curves of duration of response in all responded patients after CD7 CAR T-cell therapy. B Kaplan-Meier curves of duration of response in subgroups according to SCT after CD7 CAR T-cell therapy. Fig. S5. Representative staining of T, NK and NKT cells in the peripheral blood of a patient. Fig. S6. CD4+ and CD8+ T cell ratio in the peripheral blood of all patients. Fig. S7. Characterization of CD7- T cells from patients post CD7 CAR T-cell therapy. A CD45RA, CD62L and CD95 expression on CD4+ and CD8+ T cells from E018 at month 20 post CD7 CAR T-cell therapy and a healthy donor, as determined by flow cytometry. B T cell receptor (TCR) sequencing data, including the sum of the sequencing reads, TCR clones reconstructing from the sequencing reads and shannon entropy as an index of TCR Vβ usage diversity from indicated patients and healthy donors. C The number of T cells secreting IFN-γ in PBMCs from indicated patients, and healthy donors, after stimulation with phytohemagglutinin (PHA, as positive control), cytomegalovirus (CMV) or Epstein-Barr viru [file 13045_2023_1427_MOESM1_ESM.pdf]

Supplementary materials

The authors provided some additional information to readers, which has been included in this supplementary material. Supplement to: Long-Term Follow-Up of Donor-Derived CD7 CAR T-Cell Therapy in Patients With T-Cell Acute Lymphoblastic Leukemia.

Table of Contents **Page**

Supplementary methods 1

Supplementary figures

Figure S1. Severe GVHD associated with higher LDH and ferritin in blood. 14

Figure S2. The incidence and persistance of GVHD according to the degree of HLA matching. 15

Figure S3. EBV and CMV viral load in the plasma. 16

Figure S4. Kaplan-Meier curves of duration of response after CD7 CAR T-cell therapy. 17

Figure S5. Representative staining of T, NK and NKT cells in the peripheral blood of a patient. 18

Figure S6. $CD4^+$ and $CD8^+$ T cell ratio in the peripheral blood of all patients. 19

Figure S7. Characterization of $CD7^-$ T cells from patients post CD7 CAR T-cell therapy. 20

Supplementary tables

Table S1. Adverse events of special interest in all 20 treated patients and in subgroups. 21

Table S2. The degree of HLA matching and GVHD. 22

Table S3. Chimerism status after CAR T-cell infusion in all 20 treated patients. 23

References 25

TRIAL DESIGN

Trial Overview, Dose and Participant Run-In

A first-in-human (FIH), single-center, single-arm, open-label, non-randomized, phase I clinical trial was designed to assess the safety and efficacy of donor-derived CD7 chimeric antigen receptor (CAR) T cells in relapsed or refractory T-cell acute lymphoblastic leukemia (r/r T-ALL) patients. The Institutional Review Board (IRB) in Beijing Gobroad Boren Hospital gave its approval to the protocol.

This trial employed a 3+3 design for safety run-in. If two or more dose-limited toxicities (DLTs) were observed in the first three to six patients, the therapy would be deemed excessively toxic and the study would be terminated for safety reasons. Based on our previous clinical experience and the limitation in manufacturing CD7 CAR T cells, the study was designed a single infusion of at target dose of 1×10^6 (± 30%) cells per kilogram of body weight (/kg). Patients will receive 5×10^5 (± 30%) CAR T cells/kg if the produced cells could not meet the target dose. The 3+3 design was used here primarily for the purpose of safety monitoring, which will stop the study if 2 or more DLTs are observed in the first 3 or 6 patients. No dose-escalation was planned in the trial.

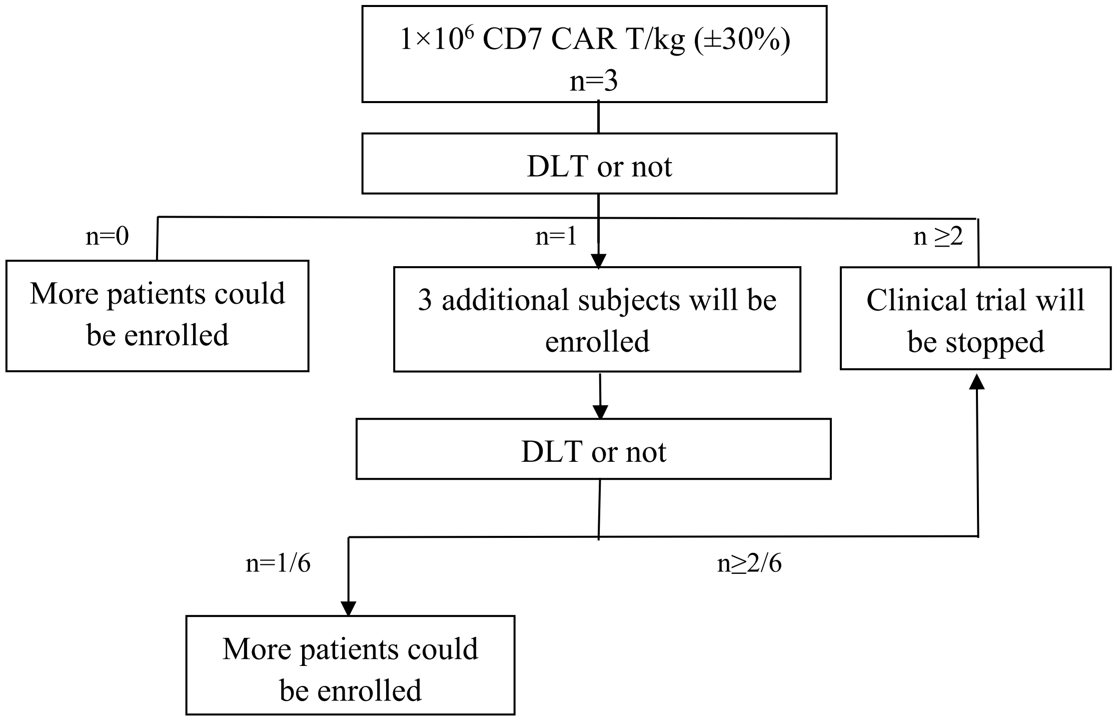

Instruction:

This study will employ the above procedure for safe run-in in standard dose group (1×10^6 CD7 CAR T cells/kg ± 30%).

• If none of the first 3 subjects in standard dose group experience a DLT, the standard dose will be continued.

• If two or more of the first 3 subjects in the standard dose cohort experience any DLTs, maximum tolerated dose (MTD) is surpassed and study will stop.

• If one of the first 3 subjects in the standard cohort experience a DLT, another three subjects will be allowed to enrolled in the same dose level:

a) If none of these another 3 subjects suffer a DLT, dose will be continued;

b) If 1 or more of these additional 3 subjects experience a DLT, MTD is surpassed and study will stop.

• If ≥ 2 of first 6 patients who receive low dose of 5×10^5 (± 30%)/kg experienced DLTs, trial will stop.

Inclusion Criteria

In order to be eligible to participate in this study, the subjects must meet all of the following criteria:

1.Candidates with relapse or refractory $CD7^+$ T cell acute lymphoblastic leukemia, who have progressed on after treatment with all standard therapies or intolerant of standard care, have limited prognosis with currently available therapies and had no available curative treatment options (such as stem cell transplantation (SCT) or chemotherapy)

2. Male or female, aged 0-70 years

3. No serious allergic constitution

4. Eastern Cooperative Oncology Group (ECOG) performance status (Oken et al., 1982) score 0 to 2

5. Have life expectancy of at least 60 days based on investigator's judgement

6. CD7 positive in bone marrow or cerebrospinal fluid (CSF) by flow cytometry, or CD7 positive in tumor tissues by immunohistochemistry (CD7 positive criteria by flow cytometry: Positive: > 80% of tumor cells expressed CD7 and the mean fluorescent intensity (MFI) of CD7 is the same as that in normal T cells; Dim: > 80% of tumor cells expressed CD7, but the MFI of CD7 is lower than that in normal T cells as least as 1 log; Partial positive: 20-80% of tumor cells expressed CD7 and the MFI

of CD7 is the same as that in normal T cells. Tumor tissue immunohistochemistry: Positive > 30% tumor cells expressed CD7)

7. Provide a signed informed consent before any screening procedure; subjects who voluntarily participate in the study should have the ability to understand and sign the informed consent form and be willing to follow the study visit schedule and relevant study procedure, as specified in the protocol. Candidates aged 19-70 years need to be sufficiently conscious and able to sign the treatment consent form and voluntary consent form. Children candidates of 0-7 can be recruited after the legal guardian or patient advocate has signed the treatment consent form and voluntary consent form. Children candidates of 8-18 years old need to be sufficiently conscious and able to sign the treatment consent form and voluntary consent form and their legal guardian or patient advocate has also need to sign the treatment consent form and voluntary consent form, respectively.

Exclusion Criteria

An individual who meets any of the following criteria will be excluded from participation in this study:

1.Intracranial hypertension or disorder of consciousness

2.Symptomatic heart failure or severe arrhythmia

3.Symptoms of severe respiratory failure

4.Complicated with other types of malignant tumors

5.Diffuse intravascular coagulation

6.Serum creatinine and/or blood urea nitrogen ≥ 1.5 times of the normal value

7.Suffering from septicemia or other uncontrollable infections

8.Patients with uncontrollable diabetes

9.Severe mental disorders

10.Obvious and active intracranial lesions were detected by cranial magnetic resonance imaging (MRI)

11.Have received organ transplantation (excluding bone marrow transplant)

12.Reproductive-aged female patients with positive blood human chorionic gonadotropin (HCG) test

13.Screened to be positive of infection of hepatitis (including hepatitis B and C), Acquired Immune Deficiency Syndrome (AIDS) and syphilis

Objectives

1. Primary Objectives

This study aimed to evaluate the safety and efficacy of donor-derived CD7 CAR T cells when administered intravenously (IV) in patients with r/r T-ALL.

2. Secondary Objectives

1) To evaluate anti-tumor activity and long-term adverse events of donor-derived CD7 CAR T cells in subjects with r/r T-ALL;

2) To characterize the pharmacokinetic (PK) profile of donor-derived CD7 CAR T cells in subjects with r/r T-ALL.

Endpoint

1. Primary Endpoints

DLTs within 21 days after infusion, the incidence of adverse events (AEs) and specially grade 3 to 5 severe adverse events (SAEs).

2. Secondary Endpoints

◊ Rates of patients having an objective response and of those achieving complete remission (CR) or CR with incomplete blood count recovery (CRi) with negative minimal residual disease ($\leq 10^{-4}$ nucleated cells, which is determined by flow cytometry) at day 15 and 30;

◊ CD7 CAR T cells proliferation and persistence in peripheral blood and cerebrospinal fluid.

Off-Study Criteria (No More Follow-Up Captured)

If a subject is no longer suitbale for receiving study therapy, they will be withdrawn from the study. No more data can be collected once a subject is withdrawn.

1. Ethical consideration
2. SAEs
3. Participants will benefit from other interventions
4. Poor compliance
   a. Irregularly monitoring and usage of drugs
   b. Using other anti-leukemia intervention which influences the assessment
   c. Other behaviors that can affect the trial

CLINICAL PROCEDURES AND ASSESSMENTS

CAR T-Cell Manufacture, Quality Control, and Infusion in Patients

The CD7 CAR lentivirus was manufactured in Shanghai YaKe company under good manufacturing practices (GMP) standard. Seven-day culture of CAR vector was also conducted for microbiological examination. All operating procedures followed the principle of GMP and standard operating procedure (SOP) in Beijing Boren Hospital.

On days -5, -4, -3 before infusion, lymphodepletion (cyclophosphamide at 250 mg/m^2/day and fludarabine at 30 mg/m^2/day for patients who received prior-SCT donor cells) or enhanced lymphodepletion (cyclophosphamide at 30 mg/kg/day and fludarabine at 30 mg/m^2/day for patients who received new donor cells) was performed. CD7 CAR T cells were given once intravenously on day 0 (target dose: 1×10^6 (± 30%) cells per kilogram body weight). Infusion of a lower dose of 5×10^5 (± 30%)/kg was allowed if the CAR T cells did not meet the target dose.

Assessment and Management of Adverse Events

Cytokine Release Syndrome and Neurotoxicity

The ASTCT Consensus was used to grade Cytokine release syndrome (CRS) and neurotoxicity.[1] Management of CRS and neurotoxicity, usage of Tocilizumab were conducted in accordance with National Comprehensive Cancer Network (NCCN) guideline.

GVHD

European Society for Blood and Marrow Transplantation (EBMT) consensus was used to grade graft-versus-host disease (GVHD),[2] which needed to be discriminated with CRS. After first efficacy evaluation at day 15, ruxolitinib was given to prevent GVHD. Skin, liver and intestinal rejections were evaluated according to guidelines listed in the table below. Interventions were given according to symptoms and laboratory indicators. Ruxolitinib (5 mg q12h) and methylprednisolone (0.5-2 mg/kg/day) were given according to GVHD grade. If the intervention had poor effects, mycophenolate mofetil was added at a dose of 0.25 g every 12 hours. If sCD25 rises rapidly, accompanied with the appearance of maculopapular skin rash within 1 day, Basiliximab was used. Details were listed below:

stage	Skin	Liver	Intestinal tract
1	Maculopapular rash < 25% of body surface	Bilirubin 34-50 μmol/l	> 500 ml diarrhoea/d
2	Maculopapular rash 25–50% of body surface	Bilirubin 51-102 μmol/l	> 1000 ml diarrhoea/d

3	Generalized erythroderma	Bilirubin 103-225 μmol/l	> 1500 ml diarrhoea/d
4	Generalized erythroderma with bullous formation and desquamation	Bilirubin > 255 μmol/l	Severe abdominal pain, with or without ileus

Grade	Degree of organ involvement
I	Stage 1-2 skin rash; no gut involvement; no liver involvement; no decrease in clinical performance
II	Stage 1-3 skin rash; stage 1 gut involvement or stage 1 liver involvement (or both); mild decrease in clinical performance
III	Stage 2-3 skin rash; stage 2-3 gut involvement or 2-4 liver involvement (or both); marked decrease in clinical performance IV
IV	Similar to Grade III with stage 2-4 organ involvement and extreme decrease in clinical performance

Infection

To manage Epstein-Barr virus (EBV), all patients who had the history of SCT were routinely orally given acyclovir as prophylaxis. In the patient who had EBV activation, ganciclovir (5 mg/kg q12h IV) was used continuously at the beginning of EBV activation. If the viral load was positive, the patients were treated with forscarnet soldium (60 mg/kg q8h IV) plus immunoglobulin (400 mg/kg for 5 days), and after 3 days, the virus copy number was reexamination and if the viral load was negative, the patients were treated with ganciclovir (5 mg/kg q12h IV). If the intervention effect is poor, giving second-line antiviral treatment such as rituximab (375 mg/m^2).

To manage cytomegalovirus (CMV), all patients who had the history of SCT were routinely orally given acyclovir as prophylaxis. If the viral load was positive, the patients were treated with forscarnet soldium (60 mg/kg q8h IV) plus immunoglobulin (400 mg/kg for 5 days), and after 3 days, the virus copy number was reexamination and if the viral load was negative, the patients were treated with acyclovir. If the intervention effect is poor, giving second-line antiviral treatment such as CMV immunoglobulin (neutralizing antibody titer is 673 IU/ml).

Bacterial infection needs to be distinguished with CRS in the process of CAR T-cell treatment. If fever occurred, an etiological examination for infection should be initiated. Some laboratory

indicators, such as the second increase of interleukin-6 (IL-6), could indicates the possibility of infection. Bacterial infection should be evidenced by positive blood cultures or other body fluid cultures or high-throughput gene detection of pathogens. Antibiotic therapy should be based on broad-spectrum coverage. For lactobacillus infections, we choose penicillin, third-generation cephalosporins (suppsin), and meropenem. For pneumococcal infections, we chose vancomycin and linezolid. Empiric treatment for infection is warranted in the neutropenic patient. If patients had sepsis or systemic bacteremia, intravenous antibiotic combination therapy may be considered. And more detailed site-specific evaluation and therapy refers to NCCN guidelines-prevention and treatment of cancer-related infections.[3]

Fungal infection is evidenced by positive blood cultures or other body fluid cultures, high-throughput gene detection of pathogens, G test or GM test. Antifungal prophylaxis (sulfamethoxazole) is used routinely in all patients with neutropenia. Antifungal agents, such as azoles and amphotericin B products, are selected based on the disease or treatment. Fluconazole is the first-line therapy, and voriconazole is used if fluconazole is ineffective. And site-specific evaluation and therapy is detailed in NCCN guidelines-prevention and treatment of cancer-related infections.[3]

Cytopenia

Cytopenias were managed in the expansion phase (> 30 days post-infusion). Patients who received CD7 CAR-T cells derived from a new donor underwent a subsequent allogeneic transplantation with stem cells from the same donor. The transplantation preconditioning regimen was determined by the transplantation physicians. For patients who were treated with CAR-T cells that were derived from prior-SCT donors, if they had grade 3 or higher cytopenia after 30 days, recombinant human granulocyte-macrophage colony-stimulating factor for injection (rhGM-CSF, 5 μg/kg/d, Tebao, Xiamen, China) was used. If rhGM-CSF is ineffective, positive selected $CD34^+$ stem cells were infused without preconditioning to promote recovery of cytopenia.[4] All related treatments were stopped after patients recovered to grade 2 cytopenia.

T and NK cell count in healthy children: Distribution by age

	0-3 months	3-6 months	6-12 months	1-2 years	2-6 years	6-12 years	12-18 years
T cells	(2.50-5.50)	(2.50-5.60)	(1.90-5.90)	(2.10-6.20)	(1.40-3.70)	(1.20-2.60)	(1.00-2.20)

NK cells	(0.17-1.10)	(0.17-0.83)	(0.16-0.95)	(0.18-0.92)	(0.13-0.72)	(0.10-0.48)	(0.07-0.48)

Number of cells per microliter × 10^{-3}, data was from Lymphocyte subsets in healthy children from birth through 18 years of age: the Pediatric AIDS Clinical Trials Group P1009 study.[5]

Assessment of Disease Burden at Baseline and post CAR T-Cell Infusion

All patients underwent bone marrow (BM) assessment by both morphological examination on smears, and flow cytometry method at baseline, and on day 15, 30 after infusion and monthly afterwards to determine the response and remission status. Cerebralspinal fluid was evaluated by flow cytometry on day 30, and whenever necessary. Imaging examinations including computed tomography (CT), position-emissiontomography (PET/CT), magnetic resonance imaging (MRI), and B-ultrasonography, were used for evaluating extramedullary diseases (EMDs) on day 15, 30 after infusion and monthly afterwards. CR or CRi, minimal residual disease (MRD), and disease relapse were assessed by the NCCN guidelines, version 1.2020.[6]

MRD^{-} was identified by flow cytometry as the undetectable leukemia cells in BM. The detection antibody was as same as the previous paragraph mentioned. The detection methods has been mentioned in the above paragraph. The MRD analyses have a sensitivity of 0.01% in our laboratory according to NCCN guidelines, which was also used in other group.[7,8]

Patient Follow-Up and Anti-Leukemia Treatment after CAR T-Cell Treatment

Patients who received prior-SCT donor CAR-T cells did not receive any further anti-leukemia treatments. Patients who received other anti-leukemia treatments would meet the endpoint and be out of trial. Patients who were treated with CAR-T cells derived from new donors underwent a SCT consolidation at around 32 days post infusion, in the trial expanding phase. Patients who did not receive post-CAR transplantation were followed up until the last follow-up. Patients who received post-CAR transplantation were followed up until the date of transplantation, but data related to their remission and survival status continued to be collected after transplantation took place.

Next Generation Sequencing of Leukemia Sample

Genomic deoxyribonucleic acid (DNA) was extracted from CD7-negative relapsed patient's peripheral blood (PB) specimen and subjected to next generation sequencing (NGS) of exon region

of CD7 genes with Ion Torrent S5 in the Medical Laboratory of Beijing Boren Hospital and Novaseq6000 in LC-Bio Technology CO., Ltd., Hangzhou, China.

ASSAYS FOR CD7-NEGATIVE T CELLS FROM PATIENTS POST CAR T THERAPY

Phenotypic Characterization by Flow Cytometry

1-2×10^5 peripheral blood mononuclear cells (PBMCs) were collected and stained with antibodies in 4 ° C out of light for 30 min, then washed by phosphate buffer saline (PBS) and resuspended in 1% DAPI PBS solution. The following antibodies were used: CD3-Percp cy5.5, CD4-APC, CD7-PEcy7, CD8-FITC, CD45RA-BV510, CD62L-PE, CD95-APCcy7 or corresponding isotype control antibody (Biolegend). Samples were acquired on the BD CANTO II and analyzed with FlowJo version 10.4 software. CD45RA, CD62L and CD95 was used to distinguish phenotype subsets, including naïve T cells (N; $CD45RA^+/CD62L^+/CD95^-$), stem cell-like memory T cells (SCM; $CD45RA^+/CD62L^+/CD95^+$), central memory T cells (CM; $CD45RA^-/CD62L^+$), effector memory T cells (EM; $CD45RA^-/CD62L^-$), and effector memory cell re-expresses CD45RA(EMRA, $CD45RA^+/CD62L^-$).

T Cell Receptor Sequencing and Repertoire Analysis

PBMCs were obtained from healthy donors or indicated patients with written informed consents. Ribonucleic Acid (RNA) was extracted from 1 × 10^6 PBMCs sample, with the exclusion of CAR T cells and tumor cells, using RNusing GenFIND DNA extraction kit (Agencourt, Beckman Coulter, Brea, CA, USA) following the manufacturer's instructions, and subsequently subjected to T cell receptor (TCR) sequencing in Beijing Gobroad Boren Hospital (Beijing, China). TCR beta chain CDR3 regions were amplified using multiplex Polymerase Chain Reaction (PCR) with primers annealing to V and J segments and sequenced by the MGISEQ-2000 platform, and also using the Oncomine™ TCR Beta-LR Assay and sequenced in Ion Torrent S5 platforms with single end 400bp strategy. The TCR sequencing raw data was first filtered to remove the adaptor contamination and low quality reads using the Fastp software by Beijing Boren Hospital (v 0.20.1, command: -f 5 -t 5 -e 25 -u 50 -l 300). The clean reads' alignment was carried out using MiXCR software following the

guideline,[9] and clones with as least 2 reads were exported for further analysis, including clonal distribution, gene recombination, and diversity analysis conducted using VDJtools.The detailed procedure was according to the software user guide.[10]

Resampled diversity and extrapolate diversity are cloning diversity under the same valid data amount, among which resampled diversity is based on the lowest data amount and extrapolate diversity is based on the largest data amount. Shannon-WieTner index was used to represent diversity.[11]

IFN-γ ELISPOT Assay with CMV/EBV Peptides

Virus-specific responding T cells were distinguished by interferon-γ (IFN-γ) ELISPOT assay as previous described.[12,13] 1×10^5 fresh PBMCs were added to 96-well plate precoated with human IFN-γ antibody (Dakewe Biotech Co., Ltd., Shenzhen, China), then stimulated with CMV peptides pool (Mabtech , Nacka Strand, Sweden) at final concentration of 2 μg/ml or EBV peptides pool (ProImmune, Oxford, UK) at final concentration of 2 μg/ml in 37℃ for 18 h. The ELISPOT plates were analyzed by the Mabtech IRIS FluoroSpot/ELISpot reader, using RAWspot technology for multiplexing at the single-cell level. If negative control wells had >5 spots formulating units (SFU) per 10^5 PBMCs or positive control wells (phytohemagglutinin stimulation) were negative, the results were excluded from further analysis.

STATISTICAL ANALYSIS

Summary of Statistical Analysis for the Trial

Statistical analysis is done using SPSS 26 or higher version. If there is no other explanation, the original data retains one decimal place, and the mean, standard deviation, median, minimum and maximum values maintain the original data's decimal places, with no more than three decimal places at most; the upper and lower confidence interval boundaries have the same number of decimal places as the point estimate; the percentage retains one decimal place, and if the percentage is zero, this statement is ignored in the result. For continuous variables, descriptive statistics were used which included means with standard deviation or medians with minimum and maximum values. For categorical variables, numbers and percentages were provided. For continuous variables, mean with standard deviation or median with lowest and maximum values were reported; for enumeration and ranked data, the number of cases and percentage were presented; the confidence interval method is

used for most of the two-category data, and α takes bilateral 0.05; time-to-event data is often described by Kaplan-Meier method.

Safety Analysis

Safety evaluation indicators include:

1) Dose-limited toxicity (DLT);

2) The incidence and severity of treatment-related adverse events (TEAE);

3) Other safety indicators such as laboratory examination results.

Description of the pre-specified safety reports in post-infusion period and the patients included in each period as follows:

Period	Definition	Inclusion of Population
Post-infusion period	From the start of the infusion to Day 30 post-infusion	Safety set
Post-treatment expanding period	From Day 30 post-infusion to 2 years	Safety set

Efficacy Analysis

Efficacy evaluation indicators include:

1. Objective Remission Rate (ORR): Complete Remission Rate (CRR), Partial Remission Rate (PR) and Duration of Remission (DOR)

2. Progression-free Survival (PFS) and Overall Survival (OS)

Duration of Remission (DOR): The time period from when the CR or CRi response criteria are initially met until the date of recurrence or death caused on by possible malignancy is referred to as the duration of remission. If at least one patient receives SCT during CD7 CAR T cell infusion remission, the date of relapse or death after SCT (if attributed to potential cancer) is used to calculate DOR.

Progression-free survival (PFS): Progression-free survival was described as the earliest date from the first infusion of CD7 CAR T cells in patients who achieved objective remission to: death for any cause after remission or relapse. If the patient has not been relapsed and died of any reason before the cutoff data, PFS will use the date of the last disease assessment as the censoring date, which should be on or before the date of the earliest censoring event. If SCT is performed in at least 1

patient after CD7 infusion during remission, the date of recurrence or death after SCT (if attributed to underlying cancer) will be used in the calculation of PFS.

Overall survival (OS): Overall survival is the time from the first CAR T cell infusion until death from any cause. Subjects who have not died before the data cutoff date take their last contact date as the deletion date, which is defined as the last date they are known to be alive. If SCT is conducted, no censor will be done. Therefore, patients who receive SCT should also be followed for survival.

Follow-up Visits

The ***15-day end of treatment (EOT) visit*** after infusion of CD7 CAR cells should evlaluate safety and efficacy in patients.

The ***30-day EOT visit*** after infusion of CD7 CAR T cells should evaluate safety and efficacy in patients.

Approximately ***2 years after completion of CD7 CAR T cell infusion***, subjects will be followed up to collect SAEs, confirm resolution of treatment-related AEs, and review concomitant medications. If laboratory tests are required to monitor unresolved AEs, it is acceptable to retrieve assessments performed at a local medical institution to the subject.

***Disease Status Follow-up Visit*:** After CD7 CAR T-cell infusion, all subjects who had response to the treatment should be followed up by disease status. Disease assessments should be performed at day 15 and every month or necessary.

Survival Follow-up Visit: Each subject (including those with disease progression) will be followed for survival every 4 weeks (±7 days) from infusion until death or withdrawal of consent. These visits may occur by telephone, email of the subject or the subject's legal guardian.

The following method was used to calculate the follow-up time of each individual: For each individual, we observe the time of entry into the study (t1), and analysis of the available data at a final end-of-study time (t2). Time to end-of-study was regard as the follow-up time in our study= t2-t1.

Subgroup Analysis

Description of adverse events and clinical responses was based on the whole cohort and subgroups as follows:

Subgroup	Precondition Regimen	Planned dose and donor type of CD7 CAR T cells

A1	250 mg/m^2 cylcophosphamide×3 days<br>30 mg/m^2 fludarabine×3 days	1×10^6 (± 30%) (7×10^5 up to and including 1.3×10^6) prior SCT donor-derived CD7 CAR T cells/kg
A2	250 mg/m^2 cylcophosphamide×3 days<br>30 mg/m^2 fludarabine×3 days	5×10^5 (± 30%) (3.5×10^5 up to and including 6.5×10^5) prior SCT donor-derived CD7 CAR T cells/kg
B1	30 mg/kg cylcophosphamide×3 days<br>30 mg/m^2 fludarabine×3 days	1×10^6 (± 30%) (7×10^5 up to and including 1.3×10^6) new donor-derived CD7 CAR T cells/kg
B2	30 mg/kg cylcophosphamide×3 days<br>30 mg/m^2 fludarabine×3 days	5×10^5 (± 30%) (3.5×10^5 up to and including 6.5×10^5) new donor-derived CD7 CAR T cells/kg

Figure S1. Severe GVHD associated with higher LDH and ferritin in blood.
Shown is the serum LDH, and ferritin for patients at the indicated time windows after CAR T-cell infusion. LDH—lactate dehydrogenase; GVHD—graft-versus-host disease; CAR—chimeric antigen receptor.

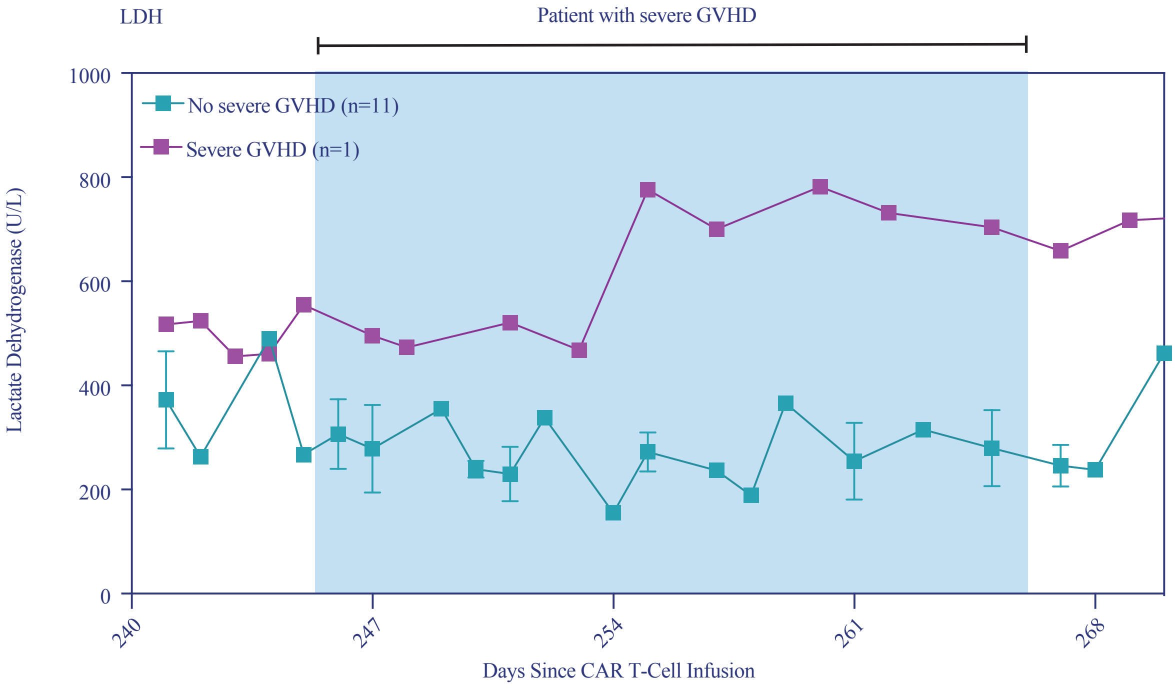

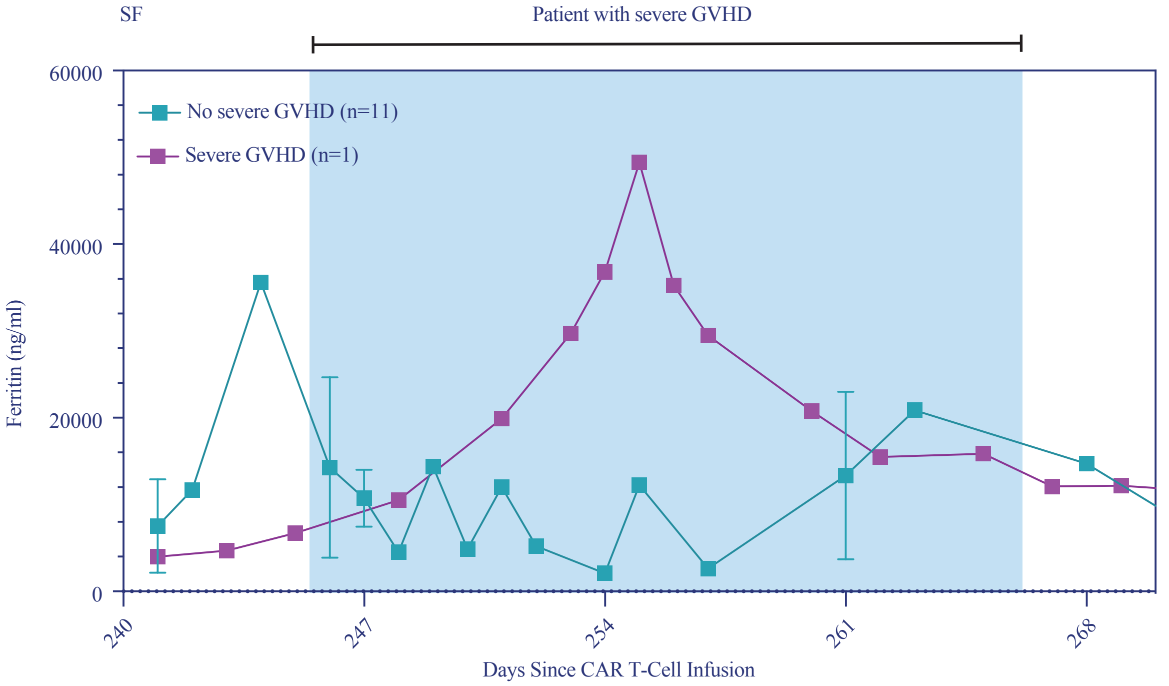

Figure S2. The incidence and persistance of GVHD according to the degree of HLA matching.
Shown the early GVHD (A) and late-onset GVHD (B) in patients receiving haploidentical donor cells and matched unrelated donor or matched sibling donor cells (MUD/MSD). Panel C and D show the persistence of early GVHD (C) and late-onset GVHD (D) between patients receiving haploidentical donor cells and MUD/MSD cells. MSD—matched sibling donor; MUD—matched unrelated donor; GVHD—graft-versus-host disease.

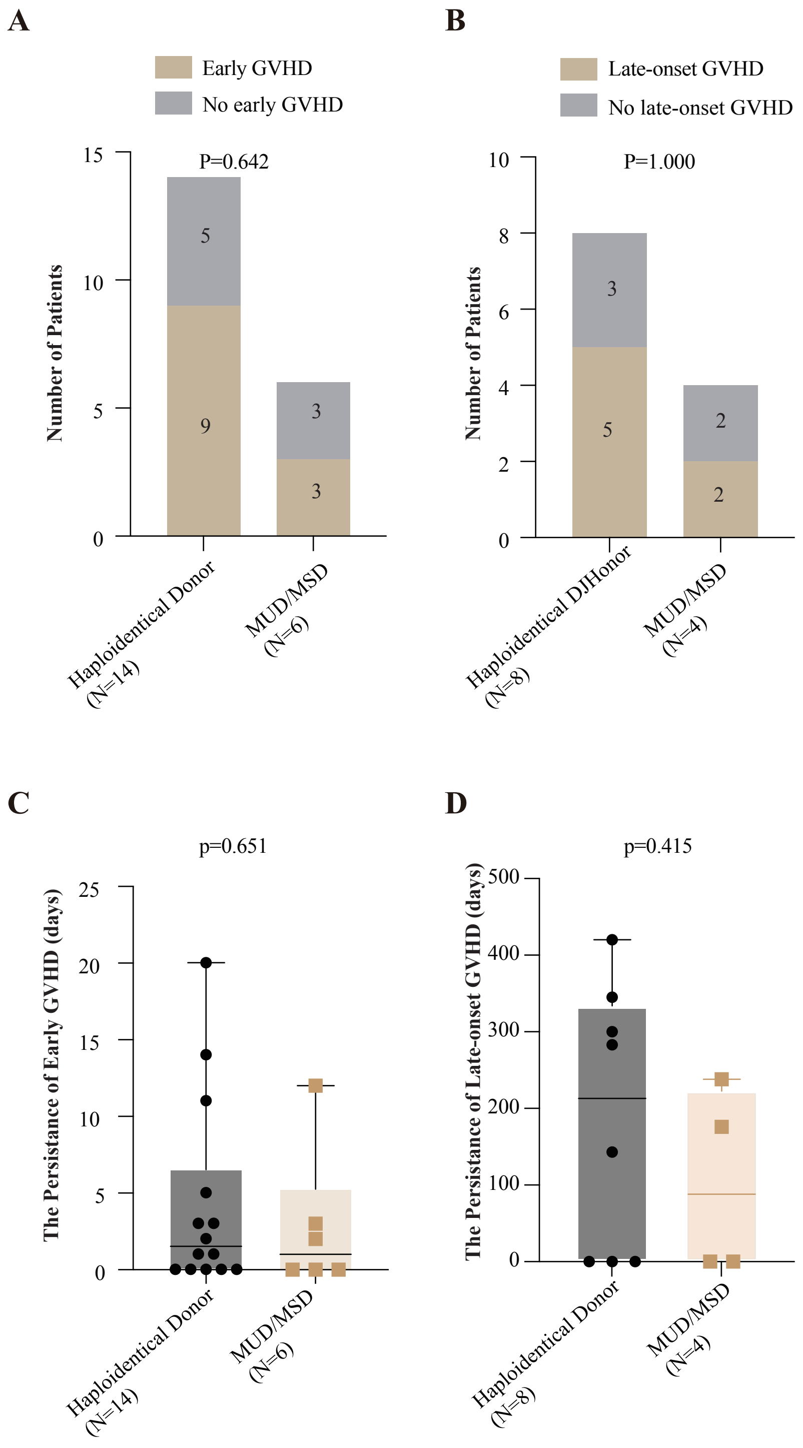

Figure S3. EBV and CMV Viral load in the plasma.
Panels A and B show the copy numbers of CMV DNA and EBV DNA in plasma of individual patients, as determined by quantitative polymerase chain reaction. Patients E010 had mixed CMV and EBV infections at 5.4 months in an outer hospital. EBV—Epstein-Barr virus; CMV—cytomegalovirus; DNA—deoxyribonucleic acid; CAR—chimeric antigen receptor.

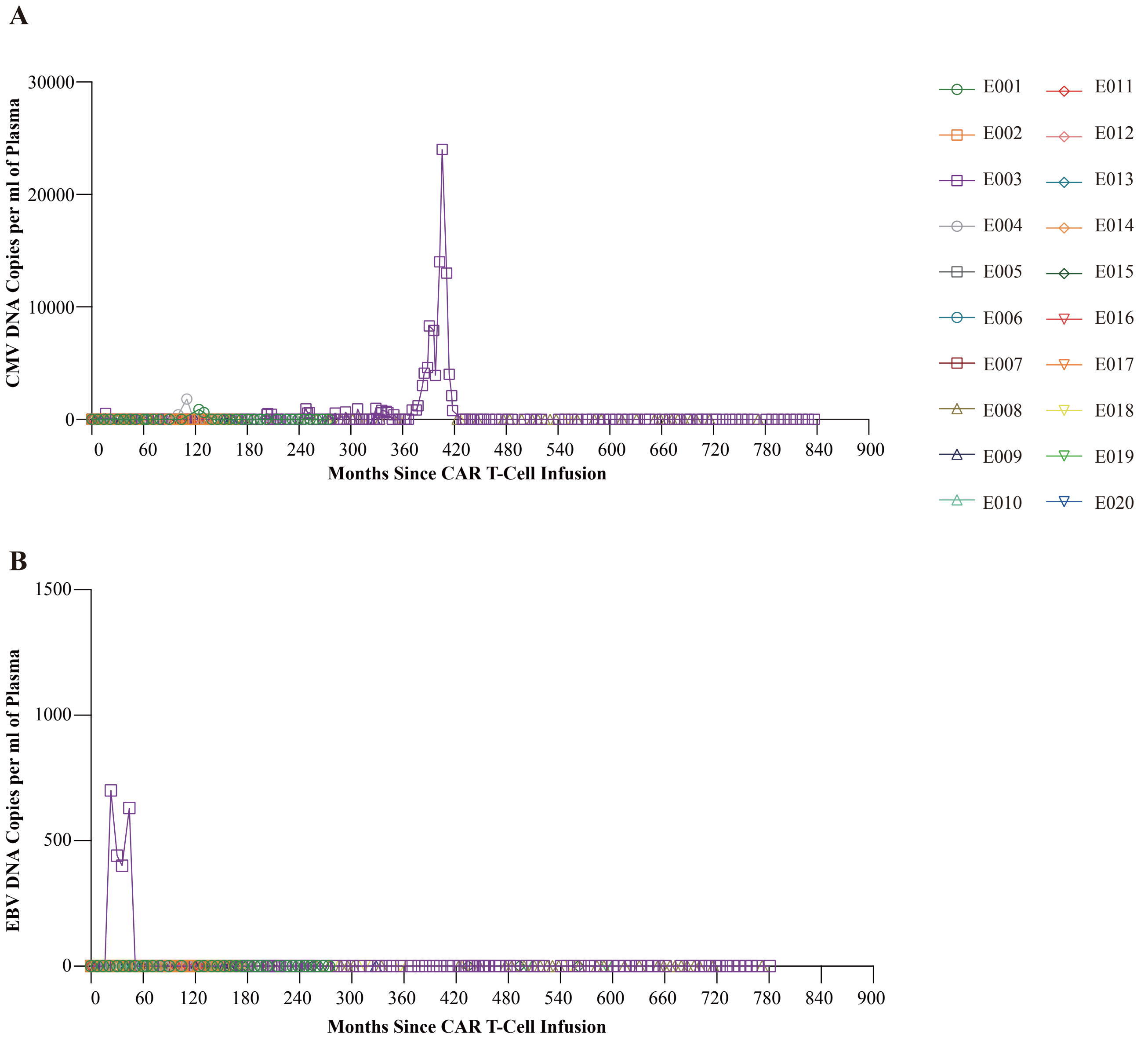

Figure S4. Kaplan-Meier curves of duration of response after CD7 CAR T-cell therapy.

Panel A shows the Kaplan-Meier curves of duration of response (DOR) in all responded patients after CD7 CAR T-cell therapy. Panel B shows the Kaplan-Meier curves of duration of response in subgroups according to SCT after CD7 CAR T-cell therapy. The time period from when the CR or CRi response criteria are initially met until the date of recurrence or death caused on by possible malignancy is referred to as the duration of remission. If at least one patient receives SCT during CD7 CAR T cell infusion remission, the date of relapse or death after SCT (if attributed to potential cancer) is used to calculate DOR. CR—complete remission; CRi—CR with incomplete blood count recovery; DOR—duration of response; SCT—stem cell transplantation; CAR—chimeric antigen receptor.

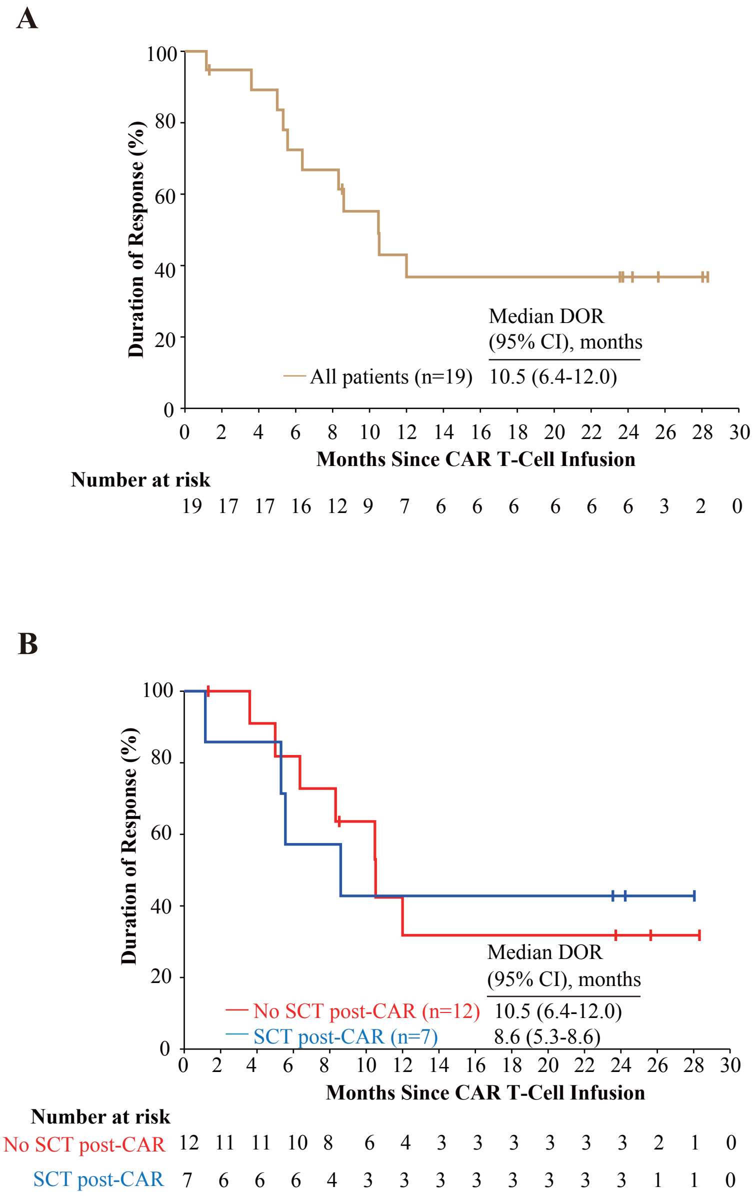

Figure S5. Representative staining of T, NK and NKT cells in the peripheral blood of a patient.
Shown the representative staining of CD7 expression on T, NK, NKT cells in the peripheral blood of patient E010 month two after CD7 CAR T cell infusion. NK—natural killer

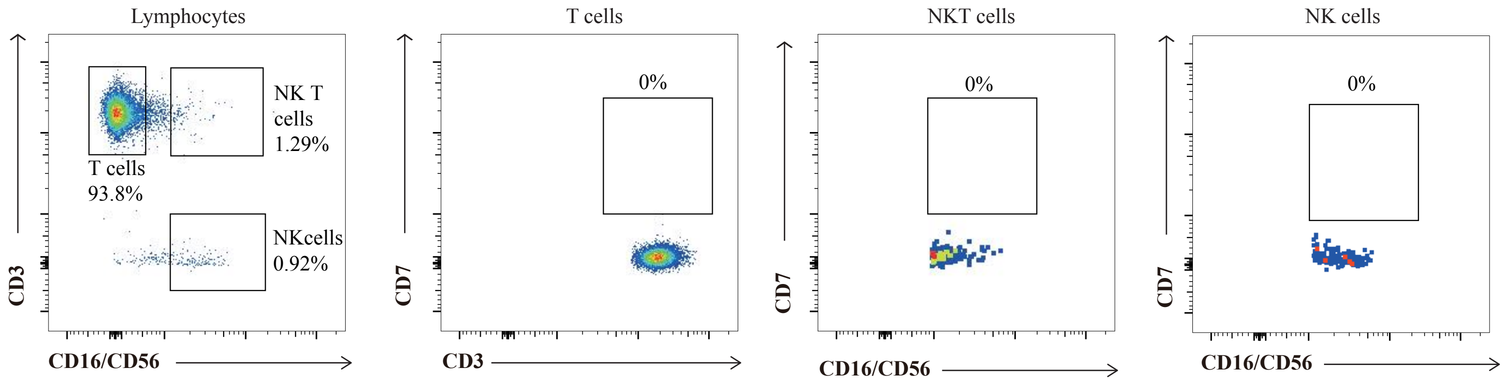

Figure S6. CD4$^+$ and CD8$^+$ T cell ratio in the peripheral blood of all patients.

Shown is the kinetics of CD4$^+$/CD8$^+$ T cells ratio in peripheral blood of all individual patients, as measured by flow cytometry. CAR T and leukemia cells are excluded in this analysis; data are recorded until the cutoff date or the time point that discontinued follow-up; different types of symbols indicate different patients, and lines of different colors indicate different subgroups.

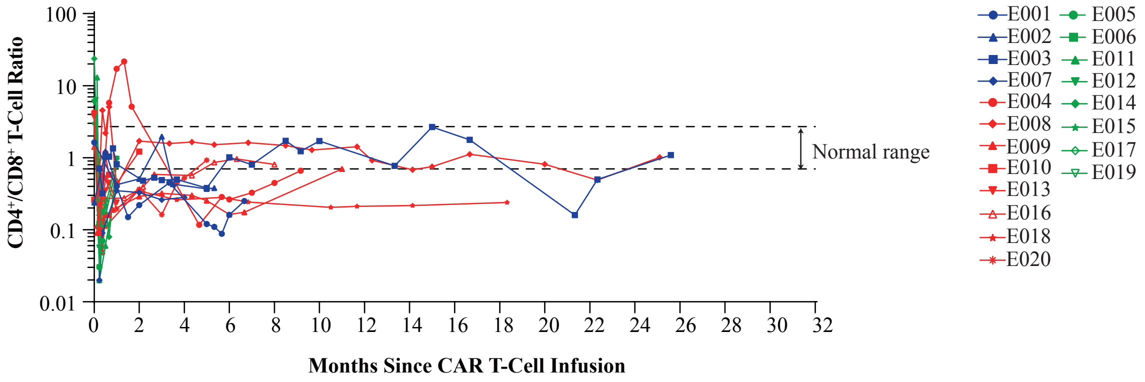

Figure S7. Characterization of CD7$^-$ T cells from patients post CD7 CAR T-cell therapy.
Panel A shows the CD45RA, CD62L and CD95 expression on CD4$^+$ and CD8$^+$ T cells from E018 at month 20 post CD7 CAR T- cell therapy and a healthy donor, as determined by flow cytometry; Panel B shows T cell receptor sequencing data, including the sum of the sequencing reads, TCR clones reconstructing from the sequencing reads and shannon entropy as an index of TCR Vβ usage diversity from indicated patients and healthy donors; Panel C shows the number of T cells secreting IFN-γ in PBMCs from indicated patients (some results had been shown in our previous articles, including E001 at month 8, E003 at month 7, E004 at month 7, E008 at month 6, E009 at month 6 and E018 at month 3), and healthy donors, after stimulation with phytohemagglutinin (PHA, as positive control), cytomegalovirus (CMV) or Epstein-Barr virus (EBV) peptide, as determined by IFN-γ ELISPOT assay.* These data had been reported in our previously report. [14]

A

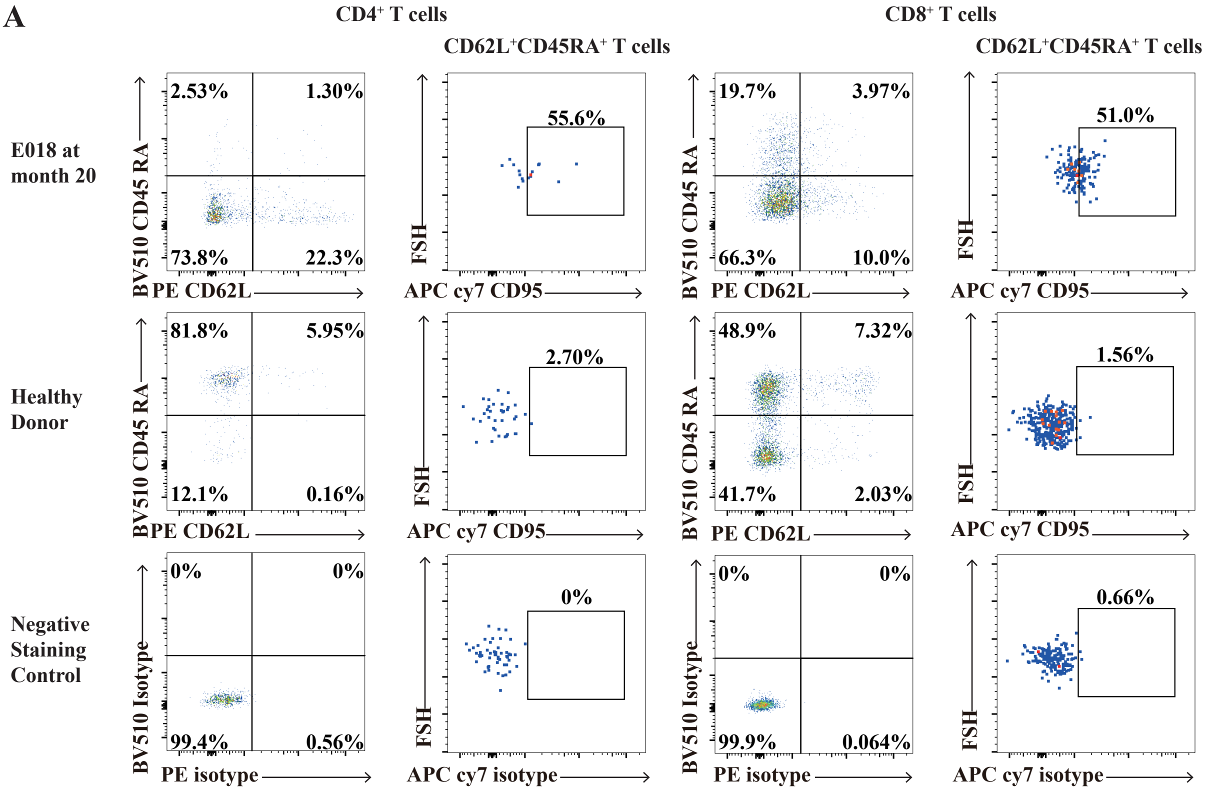

B

Independent Sample (times post last infusion)	*Reads (used)*	*Clones (total)*	*Shannon Entropy*
****E001 CAR Pt (2 months)***	7087391	33327	5.4100
E001 CAR Pt (8 months)	3384003	4608	5.0579
****E007 CAR Pt (1 months)***	7383479	24386	4.0900
****E008 CAR Pt (6 months)***	2321101	238	3.9900
E008 CAR Pt (14 months)	438520	388	2.7600
E008 CAR Pt (25 months)	659225	775	0.4046
****E009 CAR Pt (6 months)***	3032047	6067	3.3100
E018 CAR Pt (12 months)	3107818	1544	3.1158
E020 CAR Pt (4 months)	448035	1049	6.6536
Healthy Donor	251557	11387	11.2233
Healthy Donor	9201387	118380	9.9826
Healthy Donor	167827	9377	7.7540
Healthy Donor	234026	14924	10.2239

C

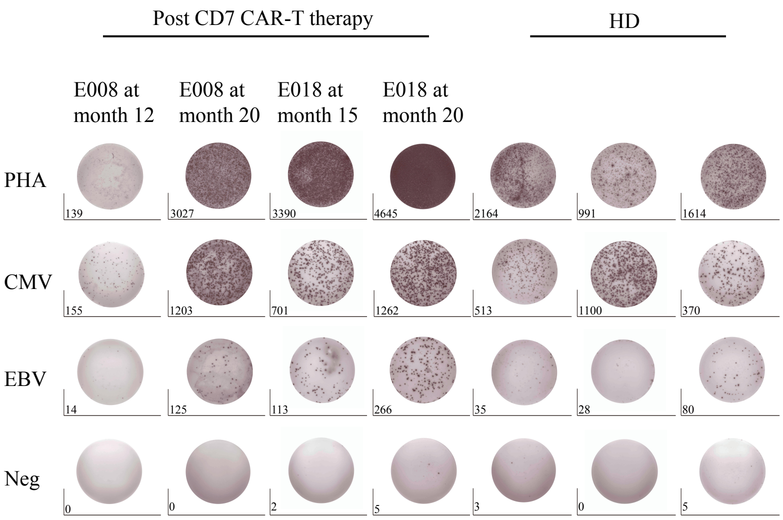

Table S1. Adverse events of special interest in all 20 treated patients and by subgroups*

	All (N=20 for AEs within day 30 and N=12 for AEs after day 30)	Prior-SCT donor, 5×10^5 (±30%)/kg (n=4)	Prior-SCT donor, 1×10^6 (±30%)/kg (n=8)	New donor, 1×10^6 (±30%)/kg (n=8)
**AEs within day 30**				
**CRS**				
Any grade	20 (100%)	4 (100%)	8 (100%)	8 (100%)
Grade 3-4	2 (10%)	1 (25%)	1 (13%)	0
Grade 5	0	0	0	0
**Neurological events**				
Any grade	3 (15%)	1 (25%)	1 (13%)	1 (13%)
Grade 3-4	0	0	0	0
Grade 5	0	0	0	0
**GVHD**				
Any grade	12 (60%)	2 (50%)	4 (50%)	6 (75%)
Grade 3-4	0	0	0	0
Grade 5	0	0	0	0
**Infection**				
Any grade	3 (15%)	2 (50%)	1 (13%)	0
Grade 3-4	0	0	0	0
Grade 5	0	0	0	0
**AEs after day 30***				
**GVHD**				
Any grade	7 (58%)	2 (50%)	5 (63%)	NA
Grade 3-4	1 (8%)	0	1 (13%)	NA
Grade 5	0	0	0	NA
**Infection**				
Any grade	6 (50%)	3 (75%)	3 (38%)	NA
Grade 3-4	1 (8%)	1 (25%)	0	NA
Grade 5	4 (33%)	1 (25%)	3 (38%)	NA

Data presented as No, (%) unless otherwise specified

*AEs after day 30 only applied to the 12 patients who received prior SCT donor-derived CAR T cells and did not receive subsequent SCT. All the 8 patients who received new donor derived CD7 CAR T cells proceeded to SCT at about day 30, therefore discontinued monitoring for AEs thereafter.

Abbreviations: AEs—adverse events; CRS—cytokine release syndrome; GVHD—graft-versus-host disease; SCT—stem-cell transplantation; NA—not applicable due to proceeding to stem-cell transplantation following CAR T-cell infusion

Table S2. The degree of HLA matching and GVHD

Donor Origin	Pt. (no.)	HLA Allelic Match (no./total no.)	Donor type	Early GVHD grade	Early GVHD duration (days)	Late-onset GVHD grade	Late-onset GVHD duration (days)
**Prior-SCT Donor, 5 × 10^5 (± 30%)/kg**	E001	6/6	MSD	0	0	1 (skin)	176
	E002	6/6	MSD	1 (skin)	12	0	0
	E003	5/10	Haploidentical donor	1 (skin)	20	1 (skin)	420
	E007	8/10	Haploidentical donor	0	0	0	0
**Prior-SCT Donor, 1 × 10^6 (± 30%)/kg**	E004	5/10	Haploidentical donor	1 (skin)	11	1 (skin)/4 (intestinal)	345/20
	E008	5/10	Haploidentical donor	2 (liver)	5	1 (skin)	283
	E009	5/10	Haploidentical donor	0	0	1 (skin)	300
	E010	5/10	Haploidentical donor	0	0	0	0
	E013	5/10	Haploidentical donor	1 (skin)	3	0	0
	E016	10/10	MSD	1 (skin)	3	0	0
	E018	10/10	MUD	0	0	1 (skin)	238
	E020	9/10	Haploidentical donor	0	0	1 (skin)	143
**New Donor, 1 × 10^6 (± 30%)/kg**	E005	5/10	Haploidentical donor	1 (skin)	14	NA	NA
	E006	5/10	Haploidentical donor	0	0	NA	NA
	E011	10/10	MSD	1 (skin)	2	NA	NA
	E012	5/10	Haploidentical donor	1 (skin)	3	NA	NA
	E014	5/10	Haploidentical donor	1 (skin)	1	NA	NA
	E015	5/10	Haploidentical donor	1 (skin)	2	NA	NA
	E017	6/10	Haploidentical donor	1 (skin)	1	NA	NA
	E019	10/10	MSD	0	0	NA	NA

Abbreviations: Pt—patient; no.—number; HLA—human leukocyte antigen; GVHD—graft-versus-host disease; MSD—matched sibling donor; MUD— matched unrelated donor; NA—not applicable for understanding the possible relationship between GVHD and CAR persistence due to proceeding to stem-cell transplantation following CAR T-cell infusion.

Table S3. Chimerism status after CAR T-cell infusion in All 20 treated patients

Donor Origin	Pt (*no.*)	Day 0 or Before Infusion*			Day 15*			Day 30*			Day 90*			Day 180*			Day 360			Day 540		
		PB T cells	BM MNCs	CAR %	PB T cells	BM MNCs	CAR %	PB T cells	BM MNCs	CAR %	PB T cells	BM MNCs	CAR %	PB T cells	BM MNCs	CAR %	PB T cells	BM MNCs	CAR %	PB T cells	BM MNCs	CAR %
**Prior-SCT Donor, 5 × 10^5 (± 30%)/kg**	E001	ND	ND	0	ND	99.6%	18.2%	ND	ND	0.7%	ND	99.7%	1.0%	ND	ND	0.4%	ND	ND	ND	ND	ND	ND
	E002	ND	ND	0	ND	ND	29.6%	ND	ND	9.5%	100%	ND	2.2%	ND	ND	ND	ND	ND	ND	ND	ND	ND
	E003	ND	ND	0	ND	ND	9.1%	ND	99.9%	6.1%	ND	ND	5.5%	ND	ND	2.9%	99.2%	98.2%	0.5%	99.8%	ND	0.7%
	E007	ND	ND	0	ND	ND	9.3%	99.9%	95.3%	0.9%	ND	ND	0.3%	ND	ND	ND	ND	ND	ND	ND	ND	ND
**Prior-SCT Donor, 1 × 10^6 (± 30%)/kg**	E004	ND	ND	0	ND	ND	26.7%	ND	ND	7.3%	ND	100%	3.3%	100%	99.6%	0.2%	100%	100%	0	ND	ND	ND
	E008	ND	ND	0	ND	ND	11.8%	ND	ND	16.2%	ND	ND	2.2%	ND	ND	1.0%	99.8%	99.3%	0.4%	ND	99.7%	0
	E009	ND	88.4%	0	ND	ND	3.1%	ND	100%	0.8%	ND	ND	0.5%	ND	ND	0.2%	ND	ND	ND	ND	ND	ND
	E010	ND	ND	0	ND	ND	15.5%	ND	99.73%	1.25%	ND	ND	0.3%	ND	ND	0.2%	ND	ND	ND	ND	ND	ND
	E013	3.9%	ND	0	98.9%	98.4%	3.2%	99.8%	98.0%	12.4%	ND	ND	ND	ND	ND	ND	ND	ND	ND	ND	ND	ND
	E016	ND	99.35%	0	100%	97.7%	6.9%	ND	ND	0.6%	100%	100%	0	ND	ND	0	ND	ND	ND	ND	ND	ND
	E018	98.2%	28.9%	0	99.9%	99.4%	9.3%	ND	ND	ND	ND	ND	0.3%	ND	100%	0.2%	100%	99.8%	0	100%	98.8%	0
	E020	ND	100%	0	ND	ND	2.9%	ND	ND	0.3%	ND	ND	0.5%	ND	ND	0.6%	ND	ND	ND	ND	ND	ND
**New Donor, 1 × 10^6 (± 30%)/kg**	E005	ND	ND	0	93.2%	20.7%	16.2%	97.5%	58.0%	5.8%	ND	ND	ND	ND	ND	ND	ND	ND	ND	ND	ND	ND
	E006	ND	ND	0	ND	ND	25.9%	ND	ND	ND	ND	ND	ND	ND	ND	ND	ND	ND	ND	ND	ND	ND
	E011	ND	ND	0	94.3%	49.7%	7.7%	56.5%	63.6%	9.1%	ND	ND	ND	ND	ND	ND	ND	ND	ND	ND	ND	ND
	E012	ND	ND	0	50.7%	3.5%	3.7%	94.8%	31.4%	29.8%	ND	ND	ND	ND	ND	ND	ND	ND	ND	ND	ND	ND

	E014	ND	ND	0	95.0%	45.0%	8.7%	97.4%	73.4%	5.2%	ND	ND	ND	ND	ND	ND	ND	ND	ND	ND	ND	ND
	E015	ND	ND	0	87.0%	35.3%	4.0%	92.3%	38.2%	3.9%	ND	ND	ND	ND	ND	ND	ND	ND	ND	ND	ND	ND
	E017	ND	ND	0	98.3%	52.9%	17.7%	88.8%	64.6%	17.7%	ND	ND	ND	ND	ND	ND	ND	ND	ND	ND	ND	ND
	E019	ND	ND	0	76.8%	24.8%	4.7%	35.1%	1.6%	5.2%	ND	ND	ND	ND	ND	ND	ND	ND	ND	ND	ND	ND

All patients were assessed for chimerism rate in peripheral blood or bone marrow. Chimerism rates of ⩾95% were regarded as complete chimerism status in our center. CAR % was the percentage of CD7 CAR T cells in lymphocytes.

Abbreviations: Pt—patient; no.—number; ND—not determined; CAR—chimeric antigen receptor; PB—peripheral blood; BM—bone marrow; MNCs—mononuclear cells.* PB and BM chimerism data in these time points had been reported in our previously work.[14]

REFERENCES

1. Lee DW, Santomasso BD, Locke FL, et al: ASTCT Consensus Grading for Cytokine Release Syndrome and Neurologic Toxicity Associated with Immune Effector Cells. Biol Blood Marrow Transplant 25:625-638, 2019
2. Przepiorka D, Weisdorf D, Martin P, et al: 1994 Consensus Conference on Acute GVHD Grading. Bone Marrow Transplant 15:825-828, 1995
3. National Comprehensive Cancer Network: NCCN Clinical Practice Guidelines in Oncology: prevention and treatment of cancer-related infections.
4. Platzbecker U, Ehninger G, Bornhäuser M: Allogeneic transplantation of CD34+ selected hematopoietic cells-clinical problems and current challenges. Leuk Lymphoma 45:447-453, 2004
5. Shearer WT, Rosenblatt HM, Gelman RS, et al: Pediatric AIDS Clinical Trials Group. Lymphocyte subsets in healthy children from birth through 18 years of age: the Pediatric AIDS Clinical Trials Group P1009 study. J Allergy Clin Immunol. 112:973-80.
6. National Comprehensive Cancer Network: NCCN Clinical Practice Guidelines in Oncology: Acute lymphoblastic leukemia (version 1.2020). https://www.nccn.org/professionals/physician_gls/pdf/all.pdf
7. Maude SL, Laetsch TW, Buechner J, et al: Tisagenlecleucel in Children and Young Adults with B-Cell Lymphoblastic Leukemia. N Engl J Med 378:439-448, 2018
8. Raje N, Berdeja J, Lin Y, et al: Anti-BCMA CAR T-Cell Therapy bb2121 in Relapsed or Refractory Multiple Myeloma. N Engl J Med 380:1726-1737, 2019
9. Bolotin D, Poslavsky S, Mitrophanov I, et al: MiXCR: software for comprehensive adaptive immunity profiling. Nat Methods 12:380–381, 2015
10. Shugay M, Bagaev DV, Turchaninova MA, et al: VDJtools: Unifying Post-analysis of T Cell Receptor Repertoires. PLoS Comput Biol 11:e1004503, 2015
11. Hill MO: Diversity and Evenness: A unifying notation and its consequences. Ecology 54:427-432, 1973
12. Peng Y, Mentzer AJ, Liu G, et al: Broad and strong memory CD4+ and CD8+ T cells induced by SARS-CoV-2 in UK convalescent individuals following COVID-19. Nat Immunol 21:1336-1345, 2020

13. Peng Y, Wang B, Talaat K, et al: Boosted Influenza-Specific T Cell Responses after H5N1 Pandemic Live Attenuated Influenza Virus Vaccination. Front Immunol 6:287, 2015
14. Pan J, Tan Y, Wang G, et al: Donor-Derived CD7 Chimeric Antigen Receptor T Cells for T-Cell Acute Lymphoblastic Leukemia: First-in-Human, Phase I Trial. J Clin Oncol. 39:3340-3351, 2021
